# Intrinsic Magnetic (EuIn)As Nanowire Shells with a Unique Crystal Structure

**DOI:** 10.1021/acs.nanolett.2c03012

**Published:** 2022-11-07

**Authors:** Hadas Shtrikman, Man Suk Song, Magdalena A. Załuska-Kotur, Ryszard Buczko, Xi Wang, Beena Kalisky, Perla Kacman, Lothar Houben, Haim Beidenkopf

**Affiliations:** † Department of Condensed Matter Physics, Weizmann Institute of Science, Rehovot 7610001, Israel; ‡ Institute of Physics, Polish Academy of Sciences, Aleja Lotnikow 32/46, Warsaw PL-02-668, Poland; § Department of Physics and Institute of Nanotechnology and Advanced Materials, Bar-Ilan University, Ramat Gan 5290002, Israel; ∥ Department of Chemical Research Support, Weizmann Institute of Science, Rehovot 761001, Israel

**Keywords:** (EuIn)As, mosaic structure, core−shell, nanowires, Eu inversion plane, magnetic

## Abstract

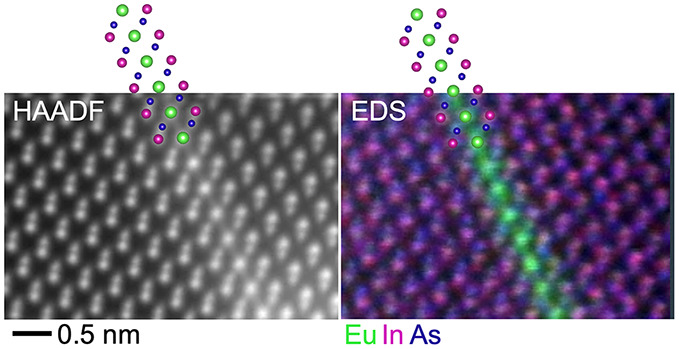

In the pursuit of magneto-electronic systems nonstoichiometric magnetic elements commonly introduce disorder and enhance magnetic scattering. We demonstrate the growth of (EuIn)As shells, with a unique crystal structure comprised of a dense net of Eu inversion planes, over InAs and InAs_1–*x*_Sb_*x*_ core nanowires. This is imaged with atomic and elemental resolution which reveal a prismatic configuration of the Eu planes. The results are supported by molecular dynamics simulations. Local magnetic and susceptibility mappings show magnetic response in all nanowires, while a subset bearing a DC signal points to ferromagnetic order. These provide a mechanism for enhancing Zeeman responses, operational at zero applied magnetic field. Such properties suggest that the obtained structures can serve as a preferred platform for time-reversal symmetry broken one-dimensional states including intrinsic topological superconductivity.

Inducing magnetism into topological electronic matter is a long-sought goal. It bears the promise to realize novel time-reversal symmetry broken topological electronic states such as the anomalous quantum Hall insulator, axion insulator, and antiferromagnetic topological insulator, each hosting unique boundary modes.^1,2^ In turn, magnetic orders can be varied via the application of a magnetic field in various orientations and temperatures, thus spanning intricate phase diagrams. Therefore, magnetic topology offers unprecedented ability to control and manipulate exotic topological boundary modes, which are well beyond increased richness.

However, the incorporation of magnetism into topologically classified materials produces a fundamental challenge, as the magnetic dopants necessarily introduce disorder. Indeed, initial attempts to dope topological insulators with magnetic elements have resulted in strong spatial fluctuations in energy and momentum across the boundaries of the doped samples.^3^ Still, conflicting evidence for time-reversal symmetry broken states has been reported. In transport measurements signatures of anomalous quantum Hall states have been found at low temperatures in magnetically doped Bi_2_Se_3_, Bi_2_Te_3_, and related ternary alloys.^4−6^ However, spectroscopically the induced time-reversal symmetry broken gap has been inconclusive.^7−10^

A different approach was taken to address the boundary modes alone by growing magnetic layers over topologically classified materials such as EuS over Bi_2_Se_3_.^11−13^ In general, those have not produced robust quantized responses. Nevertheless, zero bias conductance peaks at zero applied magnetic field, which are believed to be a sign of topological superconductivity, were observed in semiconducting InAs nanowires (NWs) with EuS half-shells.^14^

Recent efforts have thus concentrated on identifying stoichiometric compounds that are intrinsically magnetic, as well as classified as topologically nontrivial. This approach has the dual benefit of affecting the bulk topology class without introducing disorder. The classification of magnetic topological compounds is fast-growing. Prime examples are given by the ferromagnetic Weyl semimetal Co_3_Sn_2_S_2_,^15,16^ in which Fermi arc surface states have been imaged. In another antiferromagnetic topological insulator, MnBi_2_Te_4_,^17,18^ quantized Hall conductance and the lack of it were demonstrated, hallmarking the realization of anomalous quantum Hall and axion insulators, respectively.

Here we adopt a similar approach to semiconducting NWs. We grow europium indium arsenide ((EuIn)As) shells over InAs and InAs_1–*x*_Sb_*x*_ NW cores. Bulk EuIn_2_As_2_ orders antiferromagnetically below a Néel temperature of 16 K.^19^ It is a type A antiferromagnet (AFM) with As-Eu-As ferromagnetic layers that order antiferromagnetically among themselves. It was predicted to become an axion insulator within the AFM phase.^20^ We obtain an initial success toward the demonstration of intrinsically stoichiometric, magnetic semiconducting NWs. These (EuIn)As shells over InAs and InAs_1–*x*_Sb_*x*_ core NWs could serve as a superior platform for induced topological superconductivity as well as other applications that would benefit from the magnetic nature and the ability to control and manipulate it.

We start with a naive approach in which we attempted to grow a (EuIn)As shell over an InAs core NW. For this InAs core NWs were grown by molecular beam epitaxy (MBE) using the well-established gold-assisted vapor–liquid–solid (VLS) technique^21−23^ on (001)-oriented substrates.^24^ The growth of InAs NWs on a (001) InAs substrate results in wurtzite (WZ) NWs that emerge from (111) nanofacets and are thus reclining with respect to the surface. Since there are two equivalent (111) directions, the reclining NWs occasionally merge and form intersections. From the merging point of such intersections a new stalactite NW emerges, which grows vertical to the substrate and has a pure zincblende (ZB) structure.^25^ Those stalactite ZB NWs provided an important insight into the growth of (EuIn)As shells, as will be discussed below. The (EuIn)As shell was subsequently evaporated on the pregrown InAs NWs. Although desirable, in all our trials we have found only negligible (EuIn)As axial growth of NWs, which was no more than 100 nm long. Instead, (EuIn)As forms a shell around the pregrown NWs.

The resulting InAs/(EuIn)As core/shell NWs are presented in Figure 1. On all reclining WZ InAs NWs the (EuIn)As coating is rough and irregular, as demonstrated in the scanning electron microscopy (SEM) image in Figure 1a (a closer SEM view is presented in Figure SI(1)). We note that reducing the (EuIn)As thickness was not sufficient for improving the surface morphology of the (EuIn)As shell grown on a WZ core. In Figure 1b an enlarged SEM image focused on two NW intersections shows in both a rough (EuIn)As coating on the WZ arms. In striking contrast, the vertical stalactite ZB NWs extending from the intersections toward the (001) substrate have very smooth and regular (EuIn)As full shells. They attain a hexagonal cross-section, as can be seen in Figure 1c.

**Figure 1:**
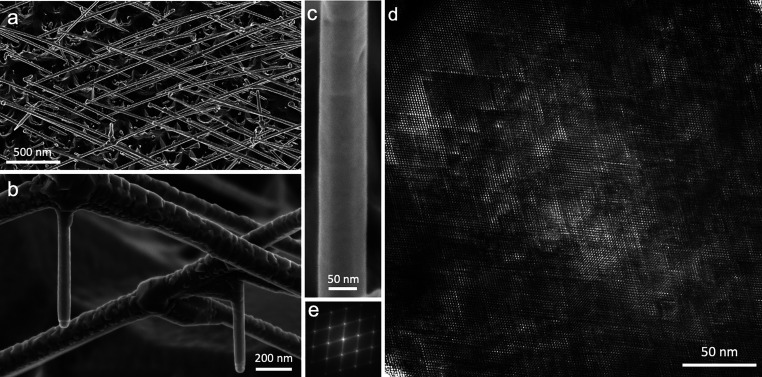
(a) Birds-eye-view SEM image of an as-grown (EuIn)As/InAs sample. (b) SEM image of two (EuIn)As/InAs NW intersections. The typical rough (EuIn)As coating on the reclining intersecting WZ InAs NWs is clearly seen. Two ZB InAs stalactite NWs emerging from the intersections are smoothly coated. (c) Higher magnification SEM image of one stalactite NW, showing the smooth surface and hexagonal shape. (d) HRTEM image of the domain boundary network seen in the (EuIn)As shell, which coats a stalactite NW. (e) Respective FT.

To resolve the crystalline structure of the (EuIn)As shells, we image them in transmission electron microscopy (TEM). An analysis of such (EuIn)As-coated WZ NWs exposes a particularly intriguing crystalline structure. The unique crystalline structure has the basic periodicity of the ZB lattice. Yet, it is composed of nanodomains separated by a high density of {111} planes. This can be seen clearly in Figure 1d and in the respective Fourier transform (FT) in Figure 1e (a TEM side view of such a single NW is shown in Figure SI(2)). The domain boundaries form a distinctive pattern in the TEM image with a network of triangular shapes in projection, which provide streaks along ⟨111⟩ directions in the FT. We find similar nanodomain structures within both the rough shells that grow on WZ cores and smooth shells that grow on the ZB stalactites.

The WZ and stalactite NWs were carefully studied by energy-dispersive X-ray spectroscopy (EDS) in order to verify the Eu composition. Eu compositions ranging from a low of few atomic percent reaching to as high as 15 atom % were found. The results for a couple of typical samples are given in Figures SI(3) and SI(4) relating to the WZ and stalactite NWs, respectively.

The regular (EuIn)As shell that grew over the ZB InAs stalactite core NWs strongly suggested that to obtain a smooth (EuIn)As coating the core should have a ZB rather than a WZ structure. To guarantee the growth of NWs with a ZB core, we have thus added a low flux of Sb during the core growth, which has been demonstrated previously to enforce a ZB structure.^26,27^ ZB InAs_1–*x*_Sb_*x*_ NWs (Sb 5–7 atom %) were grown on top of WZ InAs reclining stems. Remarkably, the ZB structure of the top InAs_1–*x*_Sb_*x*_ cores indeed enabled the formation of a smooth (EuIn)As shell, as seen in Figure 2a–c. The unique ZB structure is observed on the left-hand side of the NW in Figure 2d,e. A clear difference in the coating of the bottom WZ stem and the riding ZB structure is observed by both SEM and TEM, at the interface between the stem and the NW (pointed out by a red arrow in Figure 2c,d). The growth of (EuIn)As on the InAs_1–*x*_Sb_*x*_ ZB NWs systematically produced a smooth shell.

**Figure 2:**
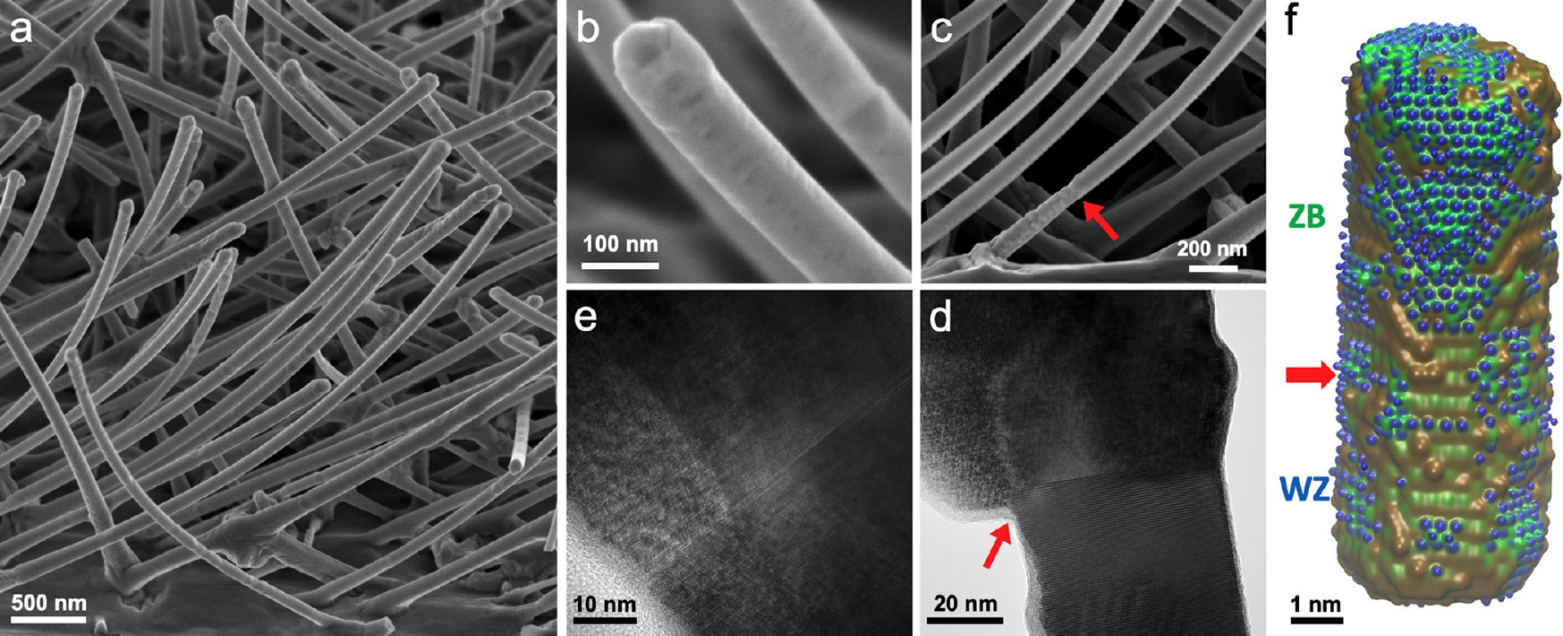
(a) A ZB InAs_1–*x*_Sb_*x*_ core improves the smoothness of the (EuIn)As shell, as substantiated by SEM of an as-grown (EuIn)As/InAs_1–*x*_Sb_*x*_ NWs sample. (b) Enlarged view of an SEM image of a single NW. Prominent differences between (EuIn)As coatings of WZ and ZB cores are apparent at the interface between the WZ stem and ZB core in (c) SEM and (d) TEM. (e) TEM image of the interface between the ZB InAs_1–*x*_Sb_*x*_ core and the (EuIn)As shell, in which a domain boundary network is clearly seen. (f) Model of an InAs NW, with a WZ structure at the bottom and ZB at the top (the interface between WZ and ZB is marked by the red arrow) obtained by molecular dynamics simulations.

Due to the reclining of the NWs grown on (001) substrates, half-shell growth also induces significant bending, which can be seen in Figure 2a–c (InAs_1–*x*_Sb_*x*_ ZB core NWs before and after (EuIn)As coating, as well as the corresponding EDS data, are presented in Figures SI(5) and SI(6), respectively). The bending of such NWs is presumably related to non-negligible lattice strain between the core and the (EuIn)As shell as well as a long migration length. It likely originates from the asymmetric evaporation of a lattice mismatched material on the reclining NWs thoroughly studied previously.^28−30^ Finally, in Figure 2f the result of modeling the formation of an (EuIn)As shell over a WZ versus ZB core NW is displayed. It shows the feasible coating of the ZB structure in contrast to the WZ structure, in perfect agreement with the experimental results.

In InAs the most advantageous positions for Eu ions incorporation are on {111} surfaces, above the triangles formed by As ions, as in the EuIn_2_As_2_ crystal.^19^ This position is available only at one polarity of the (111) surface. InAs NWs have no such surfaces as side facets, in neither the WZ nor the ZB structure. Still, seeds of such surfaces can appear in the ZB structures in steps on the {211} type facets. Since, for this favorable polarity, the positions of the Eu atoms and their distance from the arsenic layer are different from those of In atoms in InAs, in order to avoid defect formation Eu should embed into InAs over areas of the {111} surface as large as possible. To check if small {111} steps actually appear, if they can become larger as the NW grows, and thus understand why the shell with Eu ions forms much better on the ZB core structures than on WZ, the Lammps Molecular Dynamics simulation package was used to model Eu incorporation at the side facets of the NW. The initial simulated core NW has a WZ and a ZB section, and two separate Eu and As reservoirs are placed at opposite sides of the NW. While the temperature was slowly lowered, attachment of As atoms was first seen, and then Eu atoms stick to the side surfaces. The simulation proceeded until a stable core/shell NW structure was formed, as shown in Figure 2f (for calculation parameters see the Supporting Information including methods Figures SI(7) and SI(9) as well as the simulation Movie 1). Indeed, in the ZB part of the NW {2̅11}-oriented surfaces with large {111} terraces appear between the {11̅0} side facets. On such terraces regular layers of Eu can develop (see Figure SI(8)). Thus, the calculations have shown that the ZB crystal structure enables Eu incorporation and the growth of a regular shell on the NW side facets, with an atomic structure similar to that of a EuIn_2_As_2_ crystal. In the WZ structure polar surfaces of {111} type appear only in the growth direction. The WZ structure has neither such side facets nor such surfaces lying at an angle to the growth direction. Thus, the atoms which hit the NWs can only form EuAs droplets on the NW side walls.

Next, we resolve the incorporation of Eu within the (EuIn)As shells and the nanodomain structures that form in them microscopically. TEM images expose the same ZB structure comprised of a high density of nanodomains as observed in the (EuIn)As shells grown on a WZ or a stalactite InAs core. High-resolution scanning transmission electron microscope (HRSTEM) data shown in Figure 3 provide further details of the composition and structure of (EuIn)As NWs on the atomic scale. The data were obtained from (EuIn)As shells grown on ZB InAs_1–*x*_Sb_*x*_ cores. EDS chemical maps of such NWs in a side view and in a cross-section are displayed in Figures 3a,b, respectively. Diffraction data confirm the ZB structure of the InAs_1–*x*_Sb_*x*_ core and the Eu-rich shell (see Figure SI(11)). Quantification of the EDS data reveals that Eu is built into the shell with up to 12 atom %, at the expense of indium. The elemental composition of a lamella prepared from a core/shell NW with a shell of (EuIn)As grown on InAs_1–*x*_Sb_*x*_ is shown in Figure 3b and Figure SI(12). It shows a rather uniform distribution of Eu throughout the shell diameter. Similar concentrations of Eu were found also in (EuIn)As shells grown over WZ InAs cores, as well as ZB stalactite cores.

**Figure 3:**
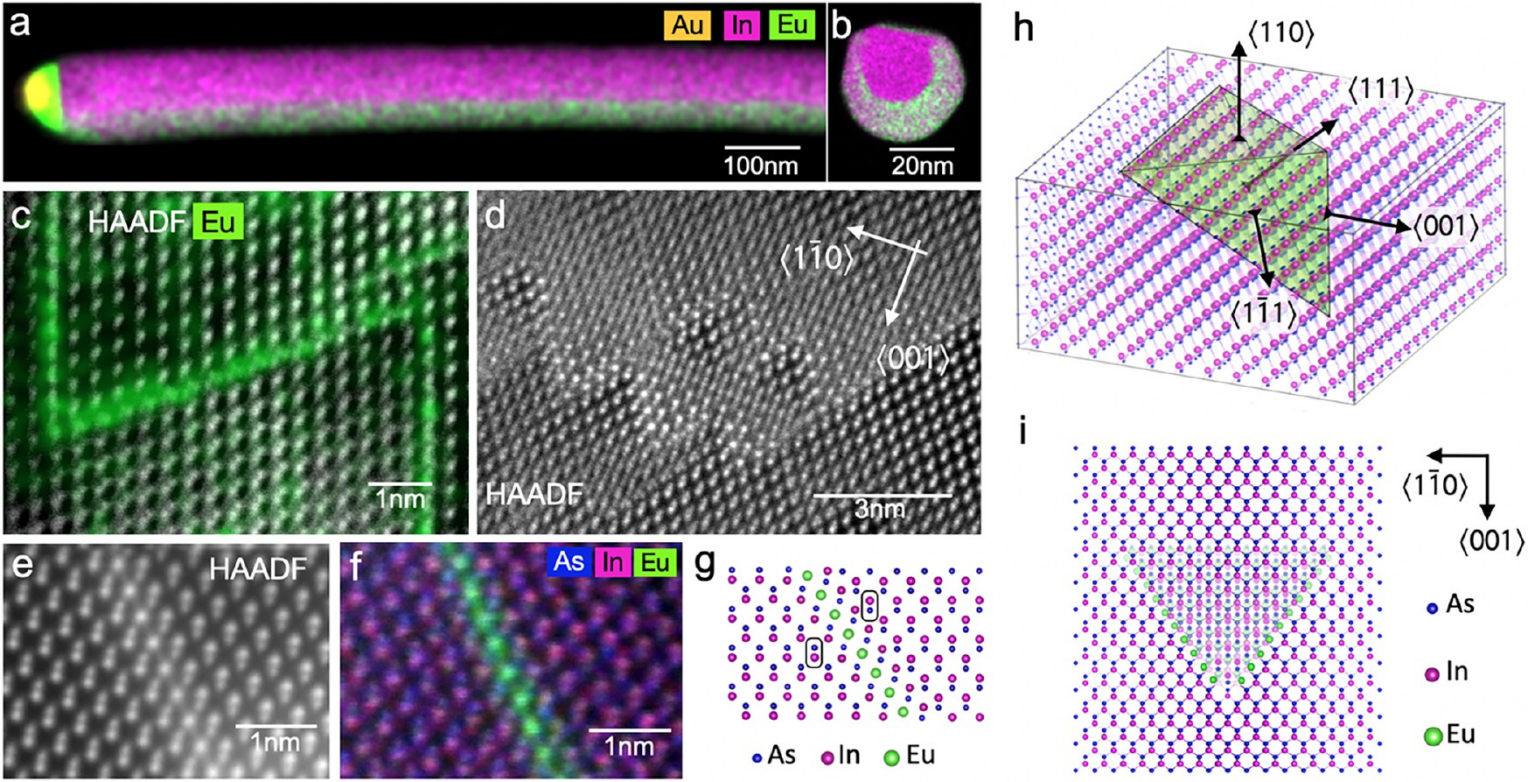
Core–shell (EuIn)As/InAs_1–*x*_Sb_*x*_ NW and the network of Eu inversion planes in the (EuIn)As shell. EDS elemental map of a core–shell (EuIn)As NW (a) in side view and (b) in cross-section. (c) HAADF image of the triangular mosaic structure with a Eu elemental map superimposed. (d) Detailed image of the triangular mosaic structure. (e) Atomic resolution image and (f) the corresponding EDS elemental map showing the inversion of the InAs lattice around the Eu inversion planes consisting of octahedrally coordinated EuAs_6_ units. (h) The 3D structure of a domain is prismatic, bound by As-terminated {111} planes. Eu planes are indicated as the green triangular faces, and the Eu atoms are left out for clarity in this graphic. (i) Top view of the atomistic model of a prismatic domain. Transparency of the atom symbols relates to the number of atoms aligned along the viewing direction: the more transparent, the fewer atoms along the line of sight.

We now focus on the triangular mosaic structure and the manner Eu incorporates in it. The triangular mosaic pattern is shown in high-angle annular dark-field (HAADF) images in Figures 3c,d. The high-angle scattering signal in a HAADF image depends sensitively on the atomic number of the scattering atoms. Hence, the stronger signal at the boundaries of the mosaic domains is associated with Eu ions. The Eu EDS signal overlaid onto the HAADF image in Figure 3c is sharply enhanced at the {111} domain boundary planes. This provides evidence that Eu ions order along these planes whose traces form the projected triangular pattern.

The atomically resolved image in Figure 3e reveals atomic sheets of Eu on {111} planes between As-terminated InAs domains. Here, the appearance of the atomic planes of Eu as chains is due to the edge-on orientation in a view along the ⟨110⟩ direction. The EDS chemical map of the same area in Figure 3f corroborates that Eu is octahedrally coordinated with As. The InAs lattice on both sides of the boundary sheet is tetrahedrally coordinated and related by point symmetry at the Eu position. This inversion of a tetrahedrally coordinated lattice by a layer of octahedrally coordinated ions strongly resembles the atomically sharp inversion domain boundaries on the basal plane of polytypoid ZnO-X_2_O_3_ compounds (X = Ga, Al, In, Fe, Sn)^31,32^ with WZ symmetry. A model of the structure of the Eu inversion planes derived from our findings is shown in Figure 3g–i. We conclude that the mosaic structure of the (EuIn)As is produced by a volume-tiling 3D network of inversion planes around Eu that condenses in 2D atomically sharp sheets in an octahedral coordination with As.

Next, we analyze how the 2D Eu inversion planes form a 3D network and eventually lead to the characteristic mosaic structure seen in the TEM images, such as in Figure 1d or 3c (see also Figure SI(13)). The full 3D structure of a single polar domain of the InAs is prismatic; its shape is determined by {111} facets and by the surface facets of the NW, when the domain cuts the surface. A 3D model of such a prismatic domain is depicted in Figure 3h. The Eu inversion planes on the (11̅1) and (1̅11) planes are oriented edge-on in a ⟨110⟩ viewing direction; they form the triangular traces in the images that are characteristic for (EuIn)As. A corresponding top view of the 3D model in the ⟨110⟩ viewing direction is shown in Figure 3i. It exposes the triangular shape. The Eu inversion planes on the equivalent (111) plane is inclined with respect to the ⟨110⟩ viewing direction; the Eu atoms on this plane give a faint signal in a TEM image, so that they remain hardly discernible. However, the existence of the Eu inversion plane on the (111) plane is evident by the trace of its interception with the surface, across which the inverted polarity of the InAs dumbbells is observed as expected. Transparency of the atom symbols in the top-view model represents the number of atoms aligning along the line of sight. The contrast of an atom symbol relates to the contrast expected in the HAADF images. Here one can infer the higher contrast at the tip of the triangular traces in HAADF, the elongated contrast of superimposed polarity domains in the viewing direction, and the seemingly reverted polarity across the trace of the intersection between the domain and the surface.

The octahedral coordination of Eu ions with As happens only on {111}B planes terminated by As. Consequently, the orientation of all prismatic domains is aligned relative to the dominant matrix orientation of the NW. This alignment of the prismatic domains is evident from images such as in Figure 1c and Figures SI(9), SI(13), and SI(14), where the apex of the triangular projection of all prismatic domains points to one common ⟨100⟩ lattice direction and hence the appearance of the pattern of scales. Since Eu creates the inversion planes, the density of the prismatic domains is a simple function of the Eu content, under the constraint of perfect termination of inverted polarity without coordination conflict.

Notably, the Eu incorporation takes place at the expense of indium in the lattice, whereas arsenic remains mostly ∼50%, as is evident from a scatter plot of EDS data across various NW samples (Figure SI(15)). EDS mapping of a lamella cross section, cut and polished from such core/shell NW, shows clearly the incorporation of the Eu in the shell, which just barely coats also the shadowed side of the NW. Occasional InAs_1–*x*_Sb_*x*_ NWs that grow vertical to the substrate have a full shell similarly to the stalactite NWs extending from the intersections downward. As mentioned above, in a few samples slight axial growth (∼100 nm) bearing a homogeneous (EuIn)As composition was observed at the NW tips, as can clearly be seen in Figure SI(16). It is related to the vertical growth taking place during the shell side growth.

Having obtained NWs with regular (EuIn)As shells, we turn to their magnetic characterization. We have employed scanning superconducting quantum interference devices (SQUIDs) operated at 4.2 K to map the local DC magnetization of the NWs. Our sensors are further equipped with a local loop that allows us to apply local AC excitation and map in addition the AC susceptibility. The core/shell (EuIn)As/InAs_1–*x*_Sb_*x*_ NWs were thus dispersed over a nonmagnetic Si/SiO_2_ substrate and measured at cryogenic temperatures. All NWs produce a clear signal in AC susceptibility (Figures SI(17)–SI(19)). Since InAs has no magnetic moment, we conclude that this strong paramagnetic response arises from the (EuIn)As shell. We note that interfaces with the InAs nanocrystallites in the As-Eu-As layer network, but also with the InAs_1–*x*_Sb_*x*_ core, may enrich the magnetic phenomenology.

Furthermore, a subset of all imaged NWs has also produced a clear signal in DC magnetization. We compare those magnetic mappings to SEM images to resolve the origin of the magnetic signals we detect. SEM images of two pairs of such NWs are shown in Figure 4a. Both of them produce strong paramagnetic signals mapped in AC susceptibility (Figure 4b). One pair (most probably a single NW of this pair) also displays a clear signal in DC magnetization, as shown in Figure 4c (see also Figures SI(17) and SI(18)). This signifies that intrinsic magnetic order is established in such (EuIn)As shells which would allow magnetic response without application of an external magnetic field. We could not identify the structural origin that distinguishes the magnetically ordered subset of (EuIn)As/InAs_1–*x*_Sb_*x*_ NWs. However, their existence suggests that intrinsic magnetism of stoichiometric NWs is within reach.

**Figure 4:**
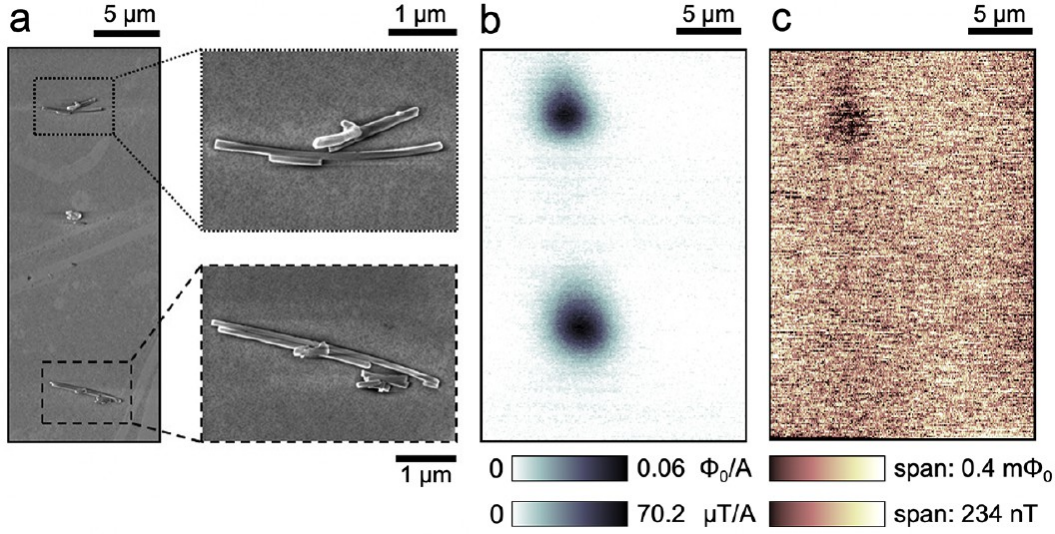
Scanning SQUID images of (EuIn)As/InAs_1–*x*_Sb_*x*_ NWs. (a) SEM images of two NW clusters, including enlarged views. (b) Local susceptibility map, demonstrating the paramagnetic response over the NWs in (a). (c) The static magnetic landscape, detecting weak ferromagnetic traces on one of the clusters. Raw data is in flux units, translated to field units by taking into account the dimensions of the scanning SQUID. Susceptibility shows the signal normalized by the field-coil current (5 mA).

Lack of a DC magnetic signal in the presence of strong AC susceptibility, which we find in the majority of the NWs, could signify either a paramagnetic state or an AFM order. Bulk EuIn_2_As_2_ has an AFM order among its As-Eu-As ferromagnetic layers. It may very well be that the Eu-As-Eu in our (EuIn)As shells are ordered ferromagnetically as well. However, the relative order of those layers is highly sensitive to the interlayer separation, which is varying within the network structure realized in our NWs. Their magnetic nature thus remains ambiguous. Nevertheless, the intrinsic paramagnetic response alone would substantially enhance the magnetic response to an externally applied magnetic field, rendering such NWs superior platforms for applications requiring magnetism.

## Supporting Information Available

Detailed experimental methods, more structural, compositional, and magnetic behavior data of (EuIn)As shells grown on WZ-InAs, ZB-InAs-stalactite, and ZB-InAs_1–*x*_Sb_*x*_ NWs comprised of SEM, HR-TEM, EDS mapping, and atomic resolution STEM-HAADF as well as scanning SQUID measurements, and additional details on the Lammps modeling performed (PDF)
Movie simulates the incorporation of Eu atoms at the side facets of a nanowire composed of a wurtzite and a zincblende section (MPG)

## Supplementary Material

nl2c03012_si_001.pdf

nl2c03012_si_002.mpg

## References

[ref1] Tokura Y.; Yasuda K.; Tsukazaki A. Magnetic Topological Insulators. Nat. Rev. Phys. 2019, 1 (2), 126–143. 10.1038/s42254-018-0011-5.

[ref2] Bernevig B. A.; Felser C.; Beidenkopf H. Progress and Prospects in Magnetic Topological Materials. Nature 2022, 603 (7899), 41–51. 10.1038/s41586-021-04105-x. 35236973

[ref3] Beidenkopf H.; Roushan P.; Seo J.; Gorman L.; Drozdov I.; Hor Y. S.; Cava R. J.; Yazdani A. Spatial Fluctuations of Helical Dirac Fermions on the Surface of Topological Insulators. Nature Phys. 2011, 7 (12), 939–943. 10.1038/nphys2108.

[ref4] Chang C.-Z.; Zhang J.; Feng X.; Shen J.; Zhang Z.; Guo M.; Li K.; Ou Y.; Wei P.; Wang L.-L.; Ji Z.-Q.; Feng Y.; Ji S.; Chen X.; Jia J.; Dai X.; Fang Z.; Zhang S.-C.; He K.; Wang Y.; Lu L.; Ma X.-C.; Xue Q.-K. Experimental Observation of the Quantum Anomalous Hall Effect in a Magnetic Topological Insulator. Science 2013, 340 (6129), 167–170. 10.1126/science.1234414. 23493424

[ref5] Hirahara T.; Otrokov M. M.; Sasaki T. T.; Sumida K.; Tomohiro Y.; Kusaka S.; Okuyama Y.; Ichinokura S.; Kobayashi M.; Takeda Y.; Amemiya K.; Shirasawa T.; Ideta S.; Miyamoto K.; Tanaka K.; Kuroda S.; Okuda T.; Hono K.; Eremeev S. V.; Chulkov E. V. Fabrication of a Novel Magnetic Topological Heterostructure and Temperature Evolution of Its Massive Dirac Cone. Nat. Commun. 2020, 11 (1), 4821 10.1038/s41467-020-18645-9. 32973165 PMC7515900

[ref6] Chang C.-Z.; Zhao W.; Kim D. Y.; Zhang H.; Assaf B. A.; Heiman D.; Zhang S.-C.; Liu C.; Chan M. H. W.; Moodera J. S. High-Precision Realization of Robust Quantum Anomalous Hall State in a Hard Ferromagnetic Topological Insulator. Nat. Mater. 2015, 14 (5), 473–477. 10.1038/nmat4204. 25730394

[ref7] Chen Y. L.; Chu J.-H.; Analytis J. G.; Liu Z. K.; Igarashi K.; Kuo H.-H.; Qi X. L.; Mo S. K.; Moore R. G.; Lu D. H.; Hashimoto M.; Sasagawa T.; Zhang S. C.; Fisher I. R.; Hussain Z.; Shen Z. X. Massive Dirac Fermion on the Surface of a Magnetically Doped Topological Insulator. Science 2010, 329 (5992), 659–662. 10.1126/science.1189924. 20689013

[ref8] Xu S.-Y.; Neupane M.; Liu C.; Zhang D.; Richardella A.; Andrew Wray L.; Alidoust N.; Leandersson M.; Balasubramanian T.; Sánchez-Barriga J.; Rader O.; Landolt G.; Slomski B.; Hugo Dil J.; Osterwalder J.; Chang T.-R.; Jeng H.-T.; Lin H.; Bansil A.; Samarth N.; Zahid Hasan M. Hedgehog Spin Texture and Berry’s Phase Tuning in a Magnetic Topological Insulator. Nature Phys. 2012, 8 (8), 616–622. 10.1038/nphys2351.

[ref9] Lachman E. O.; Young A. F.; Richardella A.; Cuppens J.; Naren H. R.; Anahory Y.; Meltzer A. Y.; Kandala A.; Kempinger S.; Myasoedov Y.; Huber M. E.; Samarth N.; Zeldov E. Visualization of Superparamagnetic Dynamics in Magnetic Topological Insulators. Science Advances 2015, 1 (10), e1500740 10.1126/sciadv.1500740. 26601138 PMC4640587

[ref10] Lee I.; Kim C. K.; Lee J.; Billinge S. J. L.; Zhong R.; Schneeloch J. A.; Liu T.; Valla T.; Tranquada J. M.; Gu G.; Davis J. C. S. Imaging Dirac-Mass Disorder from Magnetic Dopant Atoms in the Ferromagnetic Topological Insulator Crx(Bi0.1Sb0.9)2-XTe3. Proc. Natl. Acad. Sci. U. S. A. 2015, 112 (5), 1316–1321. 10.1073/pnas.1424322112. 25605947 PMC4321315

[ref11] Lang M.; Montazeri M.; Onbasli M. C.; Kou X.; Fan Y.; Upadhyaya P.; Yao K.; Liu F.; Jiang Y.; Jiang W.; Wong K. L.; Yu G.; Tang J.; Nie T.; He L.; Schwartz R. N.; Wang Y.; Ross C. A.; Wang K. L. Proximity Induced High-Temperature Magnetic Order in Topological Insulator - Ferrimagnetic Insulator Heterostructure. Nano Lett. 2014, 14 (6), 3459–3465. 10.1021/nl500973k. 24844837

[ref12] Katmis F.; Lauter V.; Nogueira F. S.; Assaf B. A.; Jamer M. E.; Wei P.; Satpati B.; Freeland J. W.; Eremin I.; Heiman D.; Jarillo-Herrero P.; Moodera J. S. A High-Temperature Ferromagnetic Topological Insulating Phase by Proximity Coupling. Nature 2016, 533 (7604), 513–516. 10.1038/nature17635. 27225124

[ref13] Hirahara T.; Eremeev S. V.; Shirasawa T.; Okuyama Y.; Kubo T.; Nakanishi R.; Akiyama R.; Takayama A.; Hajiri T.; Ideta S.; Matsunami M.; Sumida K.; Miyamoto K.; Takagi Y.; Tanaka K.; Okuda T.; Yokoyama T.; Kimura S.; Hasegawa S.; Chulkov E. V. Large-Gap Magnetic Topological Heterostructure Formed by Subsurface Incorporation of a Ferromagnetic Layer. Nano Lett. 2017, 17 (6), 3493–3500. 10.1021/acs.nanolett.7b00560. 28545300

[ref14] Vaitiekėnas S.; Liu Y.; Krogstrup P.; Marcus C. M. Zero-Bias Peaks at Zero Magnetic Field in Ferromagnetic Hybrid Nanowires. Nat. Phys. 2021, 17 (1), 43–47. 10.1038/s41567-020-1017-3.

[ref15] Morali N.; Batabyal R.; Nag P. K.; Liu E.; Xu Q.; Sun Y.; Yan B.; Felser C.; Avraham N.; Beidenkopf H. Fermi-Arc Diversity on Surface Terminations of the Magnetic Weyl Semimetal Co3Sn2S2. Science 2019, 365 (6459), 1286–1291. 10.1126/science.aav2334. 31604237

[ref16] Liu D. F.; Liang A. J.; Liu E. K.; Xu Q. N.; Li Y. W.; Chen C.; Pei D.; Shi W. J.; Mo S. K.; Dudin P.; Kim T.; Cacho C.; Li G.; Sun Y.; Yang L. X.; Liu Z. K.; Parkin S. S. P.; Felser C.; Chen Y. L. Magnetic Weyl Semimetal Phase in a Kagomé Crystal. Science 2019, 365 (6459), 1282–1285. 10.1126/science.aav2873. 31604236

[ref17] Deng Y.; Yu Y.; Shi M. Z.; Guo Z.; Xu Z.; Wang J.; Chen X. H.; Zhang Y. Quantum Anomalous Hall Effect in Intrinsic Magnetic Topological Insulator MnBi2Te4. Science 2020, 367 (6480), 895–900. 10.1126/science.aax8156. 31974160

[ref18] Liu C.; Wang Y.; Li H.; Wu Y.; Li Y.; Li J.; He K.; Xu Y.; Zhang J.; Wang Y. Robust Axion Insulator and Chern Insulator Phases in a Two-Dimensional Antiferromagnetic Topological Insulator. Nat. Mater. 2020, 19 (5), 522–527. 10.1038/s41563-019-0573-3. 31907415

[ref19] Goforth A. M.; Klavins P.; Fettinger J. C.; Kauzlarich S. M. Magnetic Properties and Negative Colossal Magnetoresistance of the Rare Earth Zintl Phase EuIn2As2. Inorg. Chem. 2008, 47 (23), 11048–11056. 10.1021/ic801290u. 18959371

[ref20] Xu Y.; Song Z.; Wang Z.; Weng H.; Dai X. Higher-Order Topology of the Axion Insulator EuIn2As2. Phys. Rev. Lett. 2019, 122 (25), 256402 10.1103/PhysRevLett.122.256402. 31347874

[ref21] Tchernycheva M.; Travers L.; Patriarche G.; Glas F.; Harmand J.-C.; Cirlin G. E.; Dubrovskii V. G. Au-Assisted Molecular Beam Epitaxy of InAs Nanowires: Growth and Theoretical Analysis. J. Appl. Phys. 2007, 102 (9), 094313 10.1063/1.2809417.

[ref22] Shtrikman H.; Popovitz-Biro R.; Kretinin A.; Heiblum M. Stacking-Faults-Free Zinc Blende GaAs Nanowires. Nano Lett. 2009, 9 (1), 215–219. 10.1021/nl8027872. 19093840

[ref23] Krogstrup P.; Yamasaki J.; Sørensen C. B.; Johnson E.; Wagner J. B.; Pennington R.; Aagesen M.; Tanaka N.; Nygård J. Junctions in Axial III–V Heterostructure Nanowires Obtained via an Interchange of Group III Elements. Nano Lett. 2009, 9 (11), 3689–3693. 10.1021/nl901348d. 19842690

[ref24] Kang J.-H.; Cohen Y.; Ronen Y.; Heiblum M.; Buczko R.; Kacman P.; Popovitz-Biro R.; Shtrikman H. Crystal Structure and Transport in Merged InAs Nanowires MBE Grown on (001) InAs. Nano Lett. 2013, 13 (11), 5190–5196. 10.1021/nl402571s. 24093328

[ref25] Kang J.-H.; Galicka M.; Kacman P.; Shtrikman H. Wurtzite/Zinc-Blende ‘K’-Shape InAs Nanowires with Embedded Two-Dimensional Wurtzite Plates. Nano Lett. 2017, 17 (1), 531–537. 10.1021/acs.nanolett.6b04598. 28002676

[ref26] Xu T.; Dick K. A.; Plissard S.; Nguyen T. H.; Makoudi Y.; Berthe M.; Nys J.-P.; Wallart X.; Grandidier B.; Caroff P. Faceting, Composition and Crystal Phase Evolution in III–V Antimonide Nanowire Heterostructures Revealed by Combining Microscopy Techniques. Nanotechnology 2012, 23 (9), 095702 10.1088/0957-4484/23/9/095702. 22322440

[ref27] Ercolani D.; Gemmi M.; Nasi L.; Rossi F.; Pea M.; Li A.; Salviati G.; Beltram F.; Sorba L. Growth of InAs/InAsSb Heterostructured Nanowires. Nanotechnology 2012, 23 (11), 115606 10.1088/0957-4484/23/11/115606. 22381938

[ref28] Gagliano L.; Albani M.; Verheijen M. A.; Bakkers E. P. A. M.; Miglio L. Twofold Origin of Strain-Induced Bending in Core–Shell Nanowires: The GaP/InGaP Case. Nanotechnology 2018, 29 (31), 315703 10.1088/1361-6528/aac417. 29749960

[ref29] Lewis R. B.; Corfdir P.; Küpers H.; Flissikowski T.; Brandt O.; Geelhaar L. Nanowires Bending over Backward from Strain Partitioning in Asymmetric Core–Shell Heterostructures. Nano Lett. 2018, 18 (4), 2343–2350. 10.1021/acs.nanolett.7b05221. 29570304

[ref30] Bjergfelt M.; Carrad D. J.; Kanne T.; Aagesen M.; Fiordaliso E. M.; Johnson E.; Shojaei B.; Palmstrøm C. J.; Krogstrup P.; Jespersen T. S.; Nygård J. Superconducting Vanadium/Indium-Arsenide Hybrid Nanowires. Nanotechnology 2019, 30 (29), 294005 10.1088/1361-6528/ab15fc. 30947145

[ref31] Schmid H.; Okunishi E.; Mader W. Defect Structures in ZnO Studied by High-Resolution Structural and Spectroscopic Imaging. Ultramicroscopy 2013, 127, 76–84. 10.1016/j.ultramic.2012.07.014. 22898248

[ref32] Remmele T.; Albrecht M.; Irmscher K.; Fornari R.; Straßburg M. Pyramidal Inversion Domain Boundaries Revisited. Appl. Phys. Lett. 2011, 99 (14), 141913 10.1063/1.3644132.

