# Supplementary material for: Intrinsic Magnetic (EuIn)As Nanowire Shells with a Unique Crystal Structure
Source: Nano Lett. 2022 Nov 7;22(22):8925–31. doi: 10.1021/acs.nanolett.2c03012 (PMC9706668; doi:10.1021/acs.nanolett.2c03012)
Supplement: Supplementary file 1 — nl2c03012_si_001.pdf [file nl2c03012_si_001.pdf]

SUPPLEMENTARY INFORMATION

Intrinsic Magnetic (EuIn)As Nanowire Shells with a Unique Crystal Structure

Hadas Shtrikman,[1]* Man Suk Song,[1] Magdalena A. Załuska-Kotur,[2] Ryszard Buczko,[2] Xi Wang,[3] Beena Kalisky,[3] Perla Kacman,[2] Lothar Houben,[4] and Haim Beidenkopf[1]

[1]*Department of Condensed Matter Physics, Weizmann Institute of Science, Rehovot 7610001, Israel*
[2]*Institute of Physics, Polish Academy of Sciences, Aleja Lotnikow 32/46, Warsaw PL-02-668, Poland*
[3]*Department of Physics and Institute of Nanotechnology and Advanced Materials, Bar-Ilan University, Ramat Gan 5290002, Israel*
[4]*Department of Chemical Research Support, Weizmann Institute of Science, Rehovot 761001, Israel*

Methods

Reclining InAs and $InAs_{1-x}Sb_x$ NWs were grown by MBE on (001) InAs using the gold-assisted vapor-liquid-solid (VLS) technique as described a couple of times prior to this work[1–3]. For the growth of $InAs_{1-x}Sb_x$ wires, the Sb shutter was opened after 1h growth on an InAs stem, and the growth was continued for another 90-100 min. Sb temperature and flux were 420 °C and 1.5×10^{-7} Torr, respectively. The growth temperature was maintained at the typical for the growth of InAs NWs temperature (~400 °C). (EuIn)As was grown immediately after growing an InAs or $InAs_{1-x}Sb_x$ core for 30 min to 120 min. The Eu temperature and flux was 450 °C and 3.5×10^{-8} Torr, respectively. Two substrate growth temperatures were investigated ~400 °C and ~465 °C**,** with no major difference.

The (EuIn)As/InAs and (EuIn)As/$InAs_{1-x}Sb_x$ NWs were characterized by field emission scanning electron microscopy (FE-SEM, Zeiss Supra-55, 3 kV, working-distance ~4 nm), transmission electron microscopy (TEM, Thermo Fisher Scientific Talos F200X, 200 kV). EDS data and mapping images were obtained by TEM with an attached detector that is identical to the one in the scanning transmission electron microscopy (STEM).

Atomic resolution TEM images and analytical data were recorded in a double aberration-corrected Themis Z microscope (Thermo Fisher Scientific Electron Microscopy Solutions, Hillsboro, USA) equipped with a high-brightness FEG at an accelerating voltage of 200 kV.

High-angle-annular dark-field (HAADF) STEM images were recorded with a Fischione Model 3000 detector with a semi-convergence angle of 21 mrad, a probe current of typically 80 pA, and an inner collection angle of 70.0 mrad. EDS hyperspectral data were obtained with a Super-X G2 four-segment SDD detector with a probe semi-convergence angle of 30 mrad and a beam current of approximately 200 pA. The EDS hyperspectral data was quantified using the Velox software (Thermo Fisher Scientific Electron Microscopy Solutions, Hillsboro, USA), by background subtraction and spectrum deconvolution. Nanobeam electron diffraction data was taken in scanning mode on an EMPAD hybrid-pixel detector with a beam semi-convergence angle of 0.19 mrad.

To examine a core/shell cross-sectional structure of NWs, lamella preparation was done using a thick lift-out procedure (Helios 600 FIB/SEM Dual Beam Microscope, Thermo Fisher Scientific). The lamella (~50 nm thick) was placed on a TEM grid and analyzed by EDS mapping in order to obtain the radial composition across the NWs diameter.
To understand why the shell with Eu ions forms much better on the ZB core structures than on WZ, the Lammps Molecular Dynamics simulation package was used to model Eu incorporation at the side facets of NW.

We used scanning superconducting quantum interference device (SQUID) microscopy to search for magnetic signals from the NWs. A SQUID functions as a magnetic flux to voltage converter, allowing sensitive detection of magnetic fields[4]. The planar SQUID, in scanning configuration, allows mapping of the static magnetic field, and the local susceptibility[5]. The sensitive area of the SQUID used in this work, the pickup loop, has a diameter of 1.5 μm. Local susceptibility is measured using an on-chip coil to apply magnetic field (the field-coil, operated at ~kHz, applies ~Gauss), while the pickup loop records the local response to the applied magnetic field. The positive signal in our data indicates a paramagnetic response. These two measurements were performed simultaneously and plotted in units of Tesla (magnetism, Phi_0 normalized by the sensitive area), and Tesla/Ampere (susceptibility, Phi_0 normalized by the sensitive area and the current in the field-coil).

(EuIn)As on WZ InAs

As discussed in the main text we launched the Europium project by evaporating (EuIn)As on the sides of reclining WZ InAs nanowires (NWs). The coating thus formed is extremely rough. Slight bending of the wires towards the substrate can be observed. In this section of the supplementary information we present a bird's eye view SEM image of an as grown sample, a TEM image of the side of a single NW and a summary of EDS data collected from an as grown NW as well as a lamella cross section prepared from such a sample. To the best of our knowledge the unique mosaic structure exposed by TEM data, was never before observed in III-V NWs.

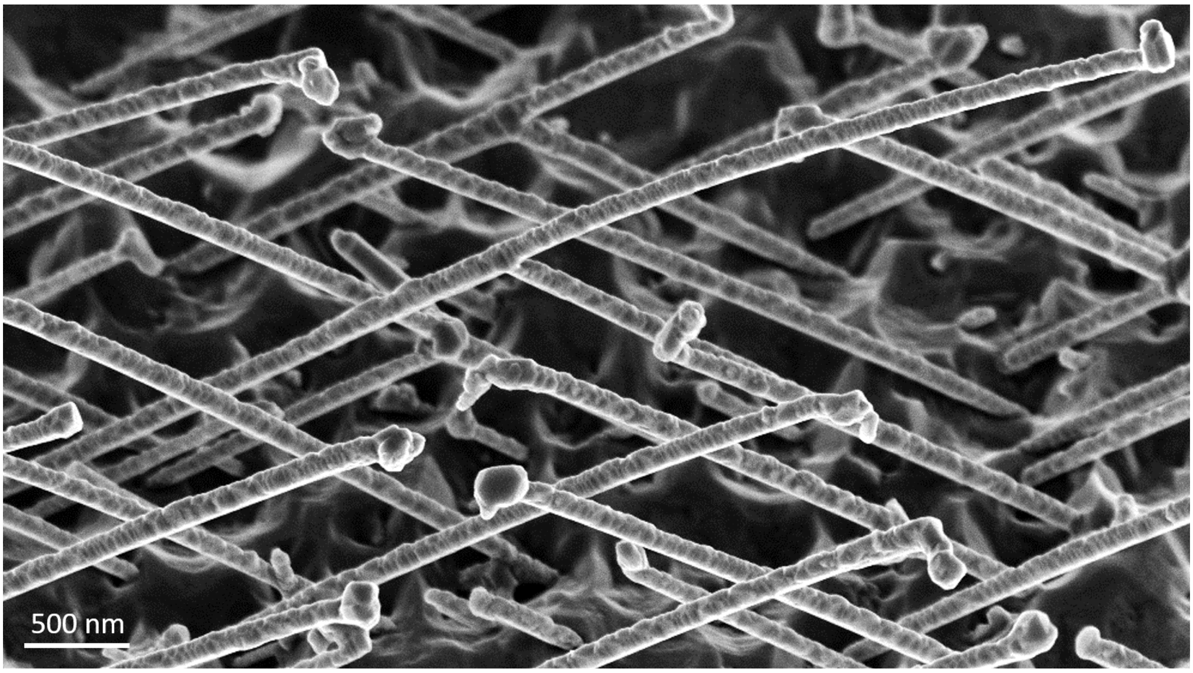

Figure SI(1): Birds eye view SEM image of as grown (EuIn)As/InAs NWs, showing slight bending towards the substrate and a rough surface.

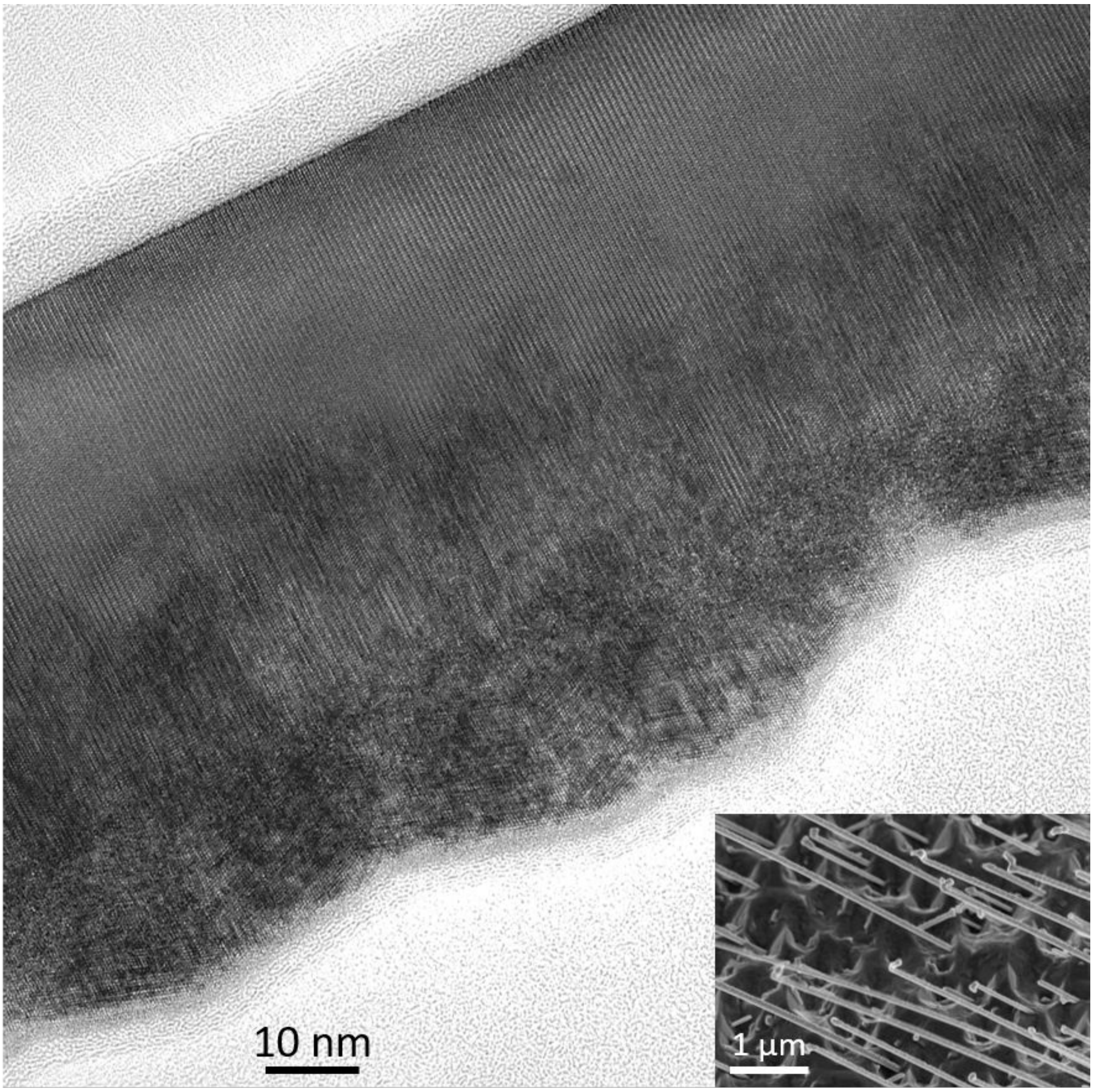

Figure SI(2): TEM image of an (EuIn)As/InAs NW exposing the unique structure of the side coating and its rough surface. The as grown sample is seen in the inset.

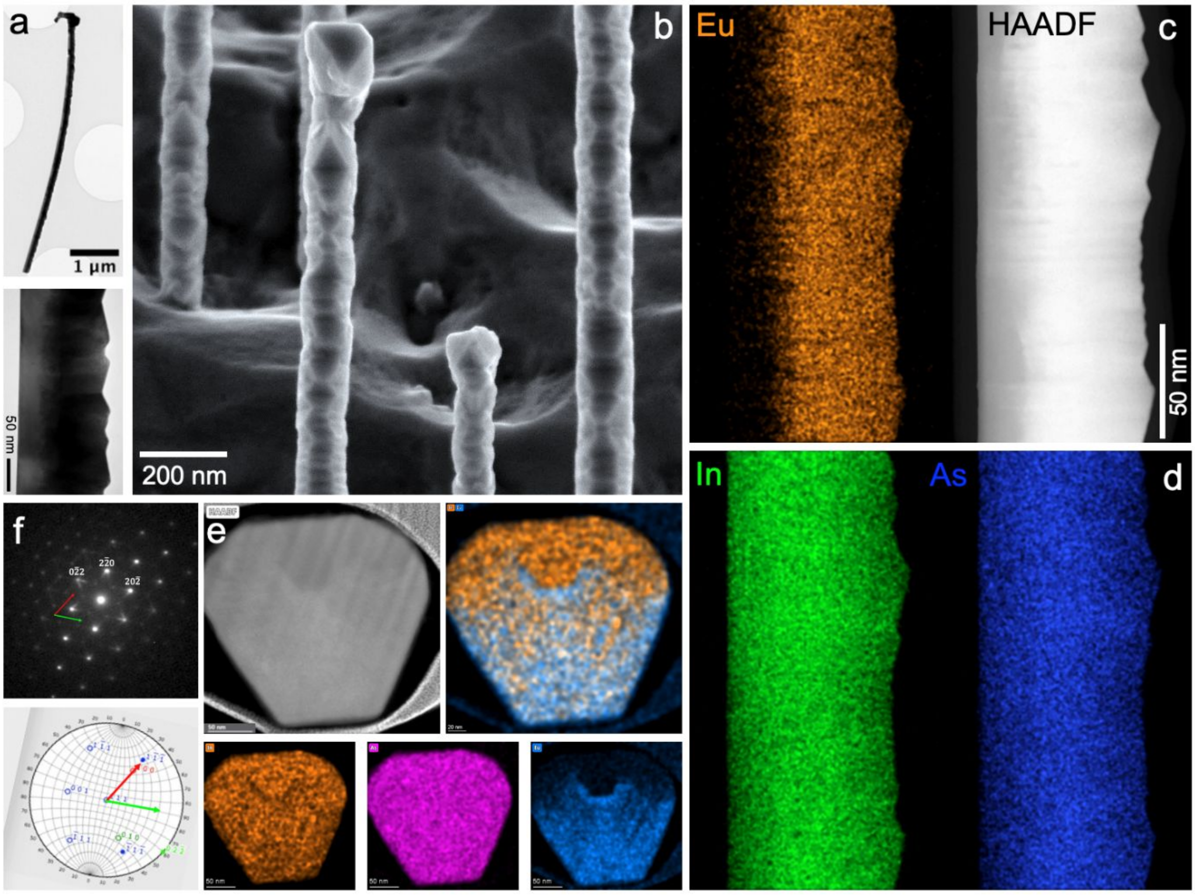

Figure SI(3): Summary of the morphology and EDS results in a typical sample comprised of a thick (EuIn)As shell grown on WZ InAs NWs.

(EuIn)As on Stalactite InAs NWs EDS Mapping

As indicated in the main text, the observation of a smooth (EuIn)As coating on vertical <001> oriented stalactite NWs formed at the intersections, was the driving force for switching from WZ to ZB core NWs[2]. This can be observed in both SEM and TEM images of the stalactite NWs, which are predominantly ZB. Yet the same structure which characterizes the (EuIn)As shell grown on WZ NWs is typical of the stalactite ones. This can be noticed even in the low magnification HADAF and TEM images. Two such images are presented in Figure SI(4) along with the respective indium, arsenic, gold and europium EDS mappings.

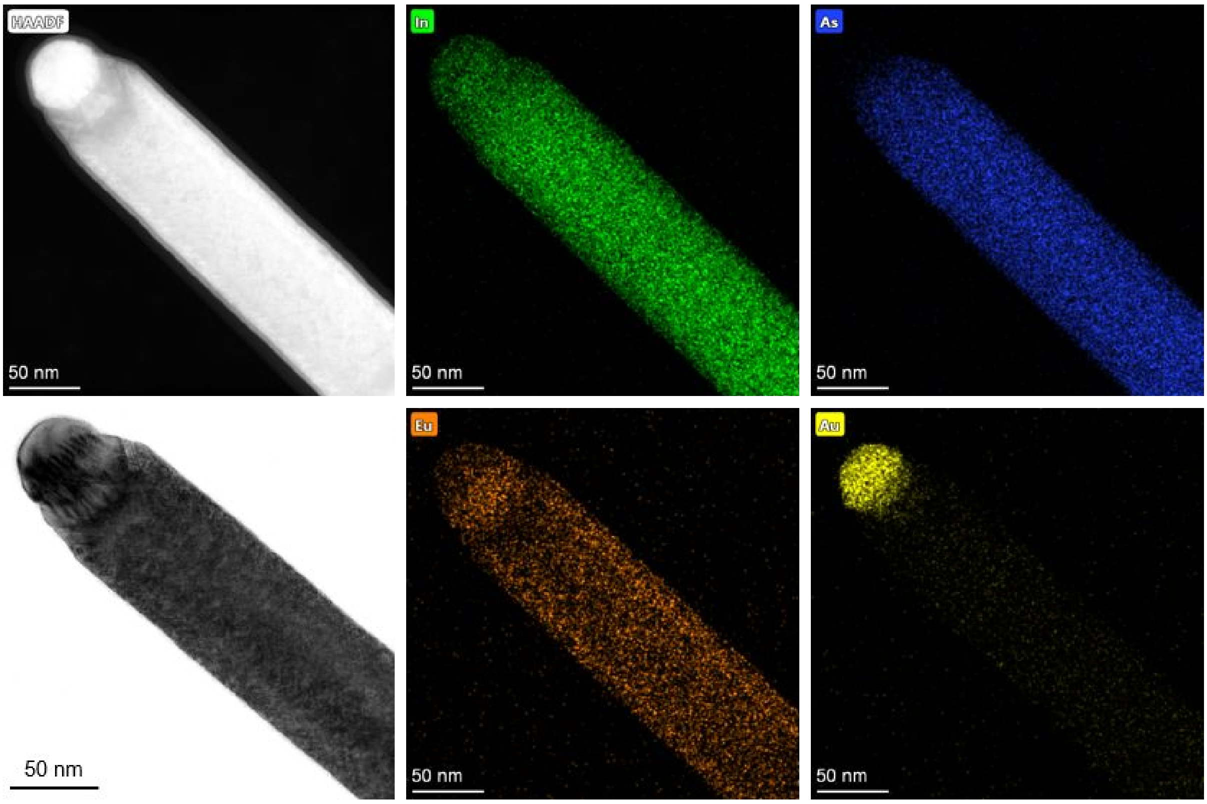

Figure SI(4): HAADF, TEM and EDS mapping of a single stalactite NW.

(EuIn)As on ZB $InAs_{1-x}Sb_x$ NWs

To improve the smoothness of the (EuIn)As shell grown on InAs we switched to growth of core NWs of $InAs_{1-x}Sb_x$ with a small concentration of antimony (5-7 atomic percent)[6,7]. This assured the ZB structure of the core NWs and their high aspect ratio and thus the smoothness of the (EuIn)As shell. In this section we add information on the ZB core $InAs_{1-x}Sb_x$ NWs and the shell formed (Figure SI(5)), with typical bending of the NWs away from the substrate. Respective EDS mappings is shown in Figure SI(6). The effect of the crystal structure of the core on the (EuIn)As is also seen in the 45° SEM image which is taken on several different core NW. This clearly shows the difference in the (EuIn)As morphology over different cores.

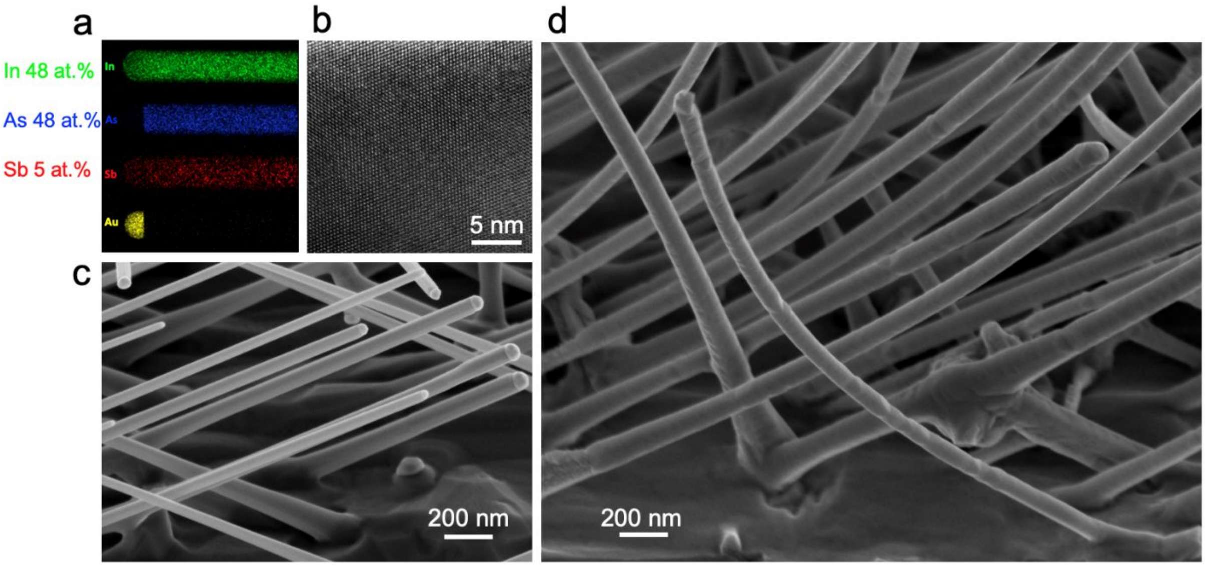

Figure SI(5): (a) EDS mapping and (b) HR-TEM of the ZB crystal structure of an $InAs_{1-x}Sb_x$ NW seen in the SEM image below (c). A 45 deg SEM image of such NWs with (EuIn)As shell is seen in (d) where the smooth coating as well as the bending towards the coated side are apparent.

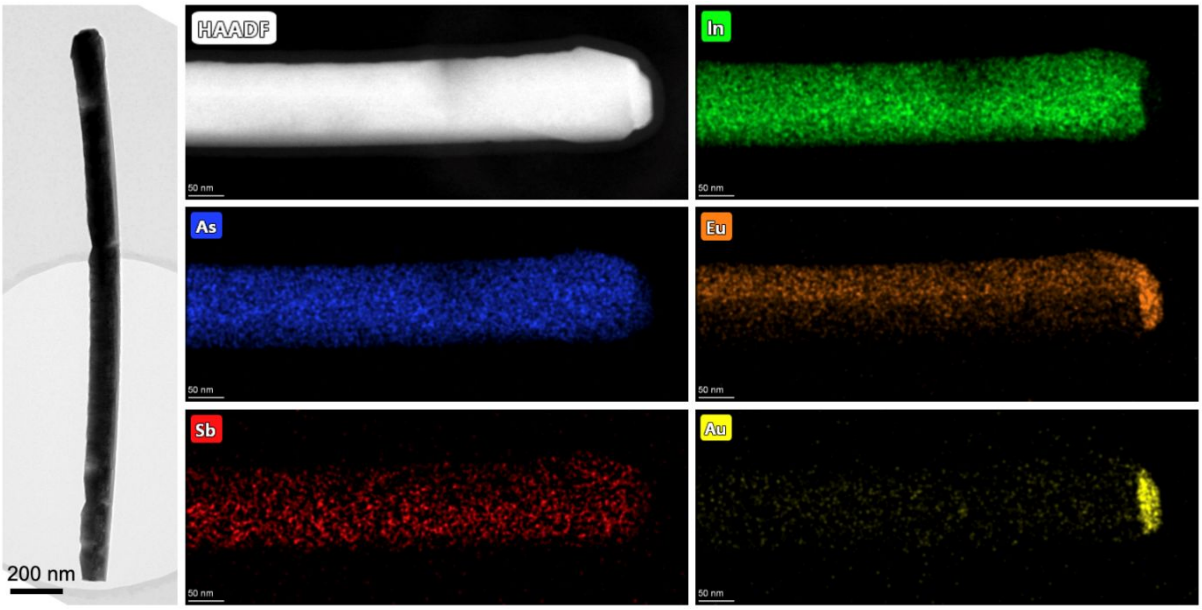

Figure SI(6): Summary of the EDS mapping of a single ZB $(EuIn)As/InAs_{1-x}Sb_x$ NW, including its low magnification TEM image which shows the bending towards the shell (note the two twinning indents).

Modeling (EuIn)As NW by molecular dynamics simulations

The core of the NW was prepared by setting In and As atoms initially at the sites of the WZ lattice. The NW was oriented in [0001] direction with six $\{1\bar{1}00\}$ side surfaces. This WZ stem was 12 InAs bilayers high. Then, [111] orientated 12 bilayers high ZB NW was positioned on top of the WZ structure. The ZB NW has also six side walls, $\{01\bar{1}\}$ oriented. The results of the simulation presented in the main body of the paper were obtained for this, shown in Figure SI(7) a, initial structure. Several other initial structures have been considered, e.g., the one with twelve side walls shown in Figure SI(7) b.

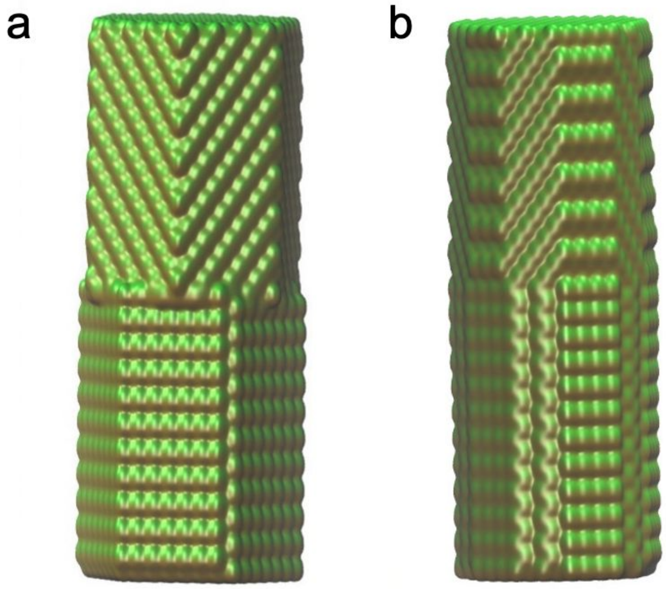

Figure SI(7): Initial core composed of 12 bilayers of WZ in the [0001] direction topped by 12 bilayers of ZB in the [111] direction. Indium atoms are brown while arsenic atoms are green. In (a) the structure has six $\{1\bar{1}00\}$ side walls and $\{01\bar{1}\}$ side walls, while in (b) another considered initial core with twelve side facets (six {110} type and six {211} type) is presented.

Two layers of As and Eu atoms were added at opposite sides of the NW, moved away from it by 30 interatomic distances. The structure was first minimized, then Lammps program was used for the Molecular Dynamics simulations. Interactions σ of Lennard-Jones 6-12 type were set between atoms. The parameters for σ reflect the length of the interatomic distance in the lattice, for ε the melting temperature. Thus, we have: σ = 2.3A for In-As interaction, 2.7A for Eu-As, and 4.2A for As-Eu, whereas for As-As, In-In and Eu-Eu pairs we used 3.8A. The depth of energy, ε, was set 0.15 eV for InAs, 0.14eV for EuAs and 0.07eV for EuIn, while all other pair interactions have strength 0.1eV. We took velocities from Boltzmann distribution at 600K, and $2\cdot10^6$ simulation steps were run at this temperature. Then, the temperature was decreased successively by 100K and in each temperature $2\cdot10^6$ simulation steps were run again. The

whole simulation was ended at 300K. The results are indeed very interesting. First, an attachment of As atoms to the NW was observed. A reconstruction of the side facets also takes place, and finally in the ZB part of the NW $\{\bar{2}11\}$ surfaces appear between the $\{1\bar{1}0\}$ oriented. Next, Eu atoms stick mainly to the side surfaces, but some of them get attached also on top or at the bottom of NW. As shown in Fig. SI(8), in contrast to the WZ $(1\bar{1}00)$ surfaces the $(\bar{2}11)$-oriented ZB surfaces contain $\{111\}$ terraces, on which the Eu atoms can attach and regular layers of Eu can develop. This is possible only for one surface polarity, which is compatible with the mutual arrangement of In and As layers in the $EuIn_2As_2$ crystal[8].

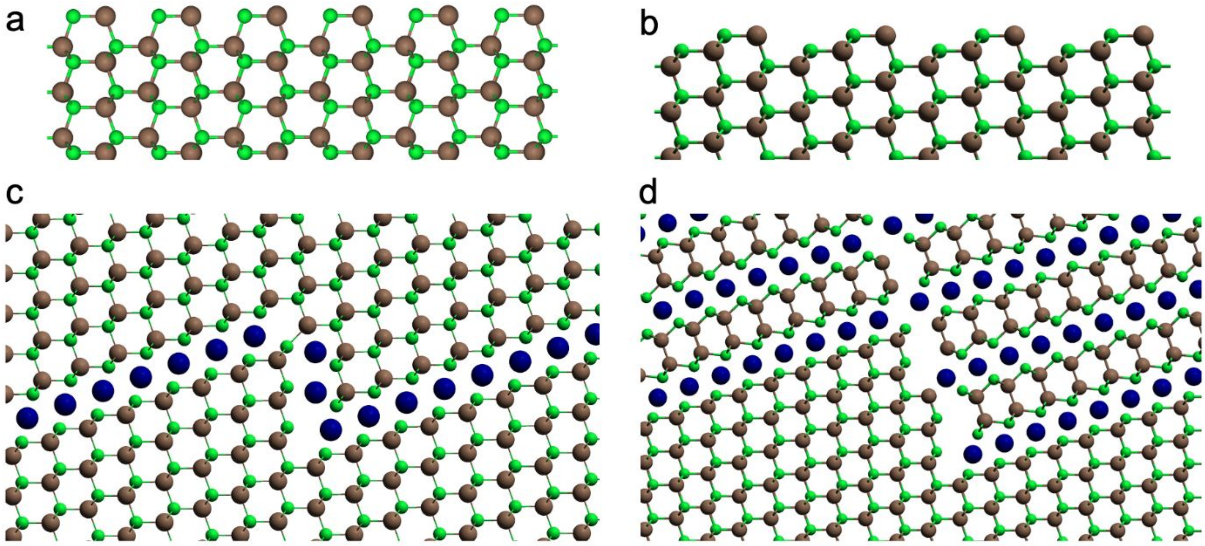

Figure SI(8): (a) (0110) WZ surface (b) (112) ZB surface (c) InAs polarity inversion at a step with 3 double atomic layers and Eu atoms (d) $EuIn_2As_2$ on InAs (112) surface with a step of 5 double atomic layers.

In order to check whether initial orientation of side walls makes a difference, another type of core NW with 12 different side facets (6 $\{1\bar{1}0\}$ and 6 $\{\bar{2}11\}$) was prepared, as shown in Fig. SI(7) b. The final effect was very similar (see Fig. SI(9) a). An impact of other conditions was also checked. Starting the simulations with 900 K caused melting of the structure and different geometry after cooling. We decided therefore to set the initial temperature to 600K. Then, several cooling procedures were tried, faster or slower, and they didn't change much the result. Finally, we checked different initial positions for the free adatoms - either closer or further to the side walls of NW but also on top and at the bottom of the NW. Again we haven't noticed much difference between the simulation results (see Fig. SI(9) b). We note only that at the beginning it was good to keep the Eu and As atoms far apart, because otherwise they tend to create crystal by themselves.

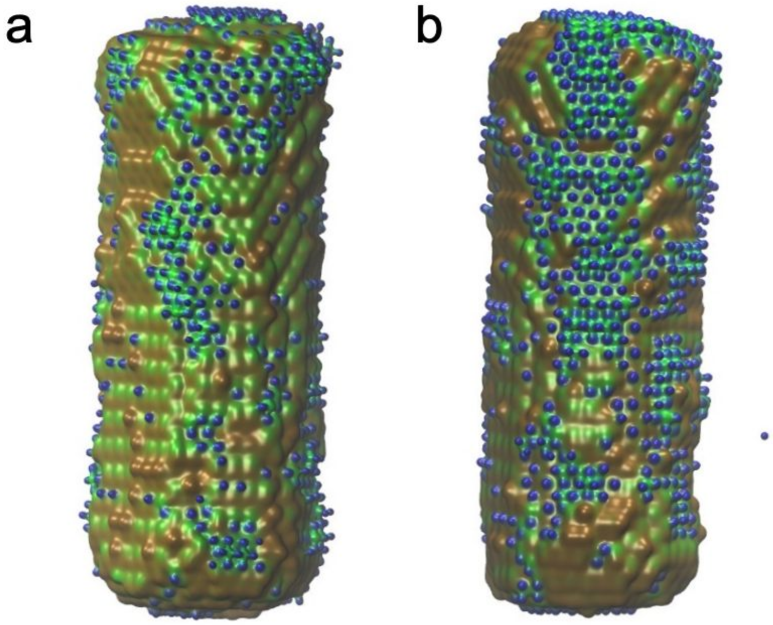

Figure SI(9): Results of the simulations performed for (a) the initial core shown in Fig. SI(7) b, i.e., with twelve side facets; (b) the initial positions for the free Eu and As adatoms at the top of the core NW with six side facets, the one shown in Fig. SI(7) a. See Movie 1.

The morphology of the (EuIn)As shell relates to the structure of the core, having a ZB or WZ structure, or single/multiple twin planes.

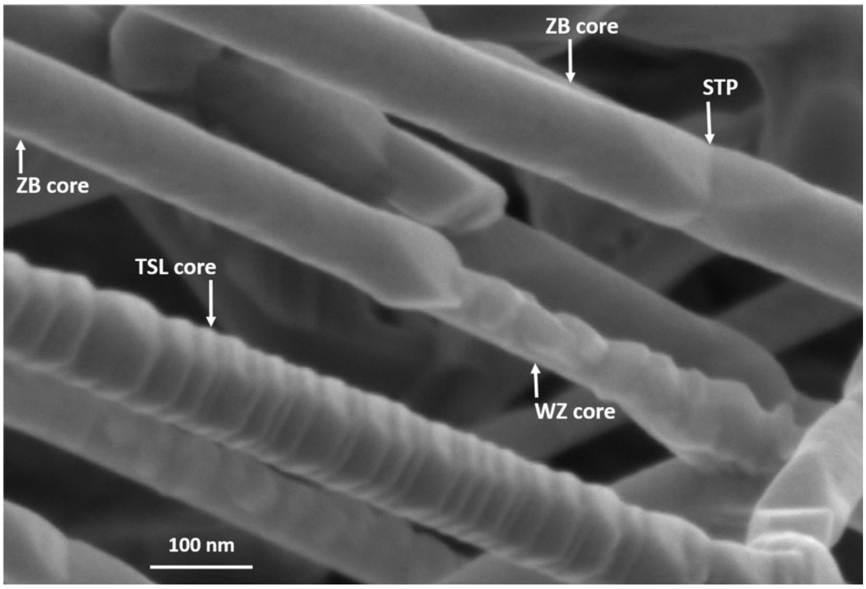

Figure SI(10): Bird's eye view image of a variety of (EuIn)As core/shell NWs emphasizing the role of the structural properties of the core. The smooth surface coating of (EuIn)As on ZB NWs is evident, as well as the rough coating of the WZ stems. The effect of single twin plane (STP) or a twinning superlattice (TSL) on the shell can be clearly seen as well.

Atomic Resolution STEM-HAADF Analysis of (EuIn)As Shells

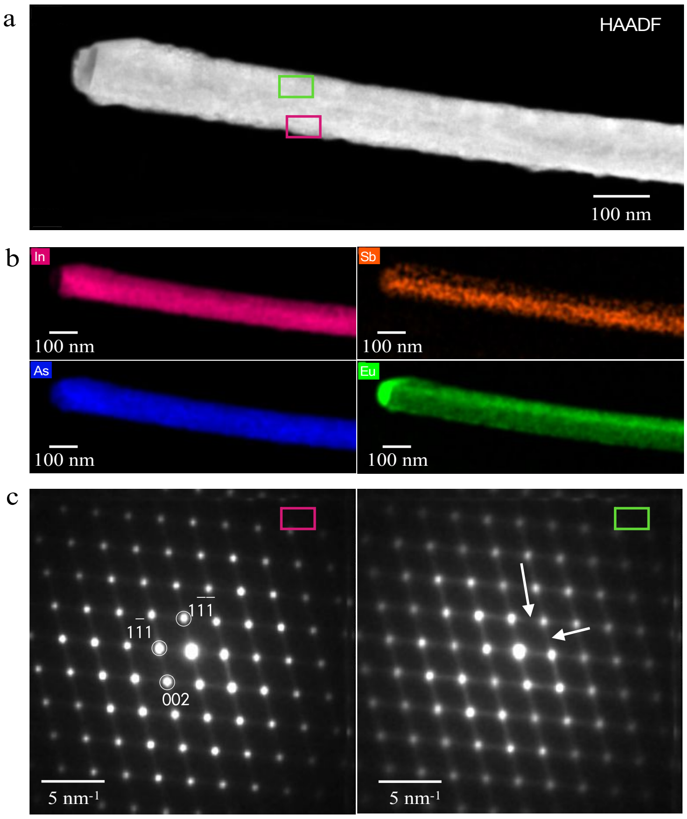

Figure SI(11): ZB structure of core/shell NWs with a shell of (EuIn)As grown on InAsSb. (a) HAADF image of a core/shell NW, with the Eu rich shell concentrating at the top part of the NW. (b) EDS elemental maps showing the distribution of In, Sb, Eu. Sb is present in the stem, in the shell Eu replaces In. (c) Electron nanobeam diffraction patterns extracted from a scanning diffraction dataset of the same NW, taken from the bottom part (left) and the top part (right), as marked in (a). Both patterns are characteristic for the <110> viewing direction of the ZB structure and indicative of the single orientation of the crystal. The prominent streaks along the two [111] directions orthogonal to the viewing direction (arrow markers) are indicative of a large density of grain boundaries on {111} plane. Note that the in the bottom part the electron beam transmits a thinner part of the shell so that faint streaks are visible in the diffraction pattern of the lower stem part as well.

a

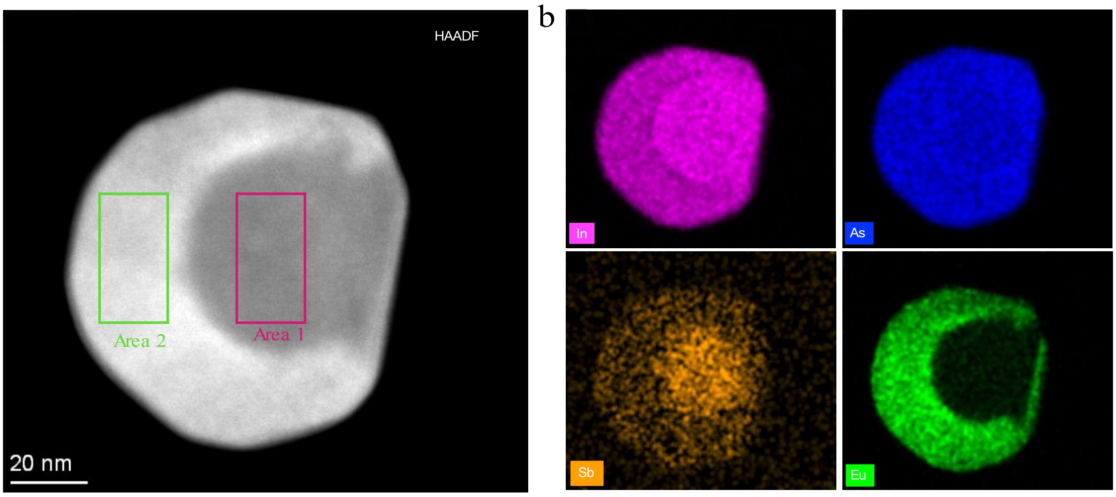

b

c

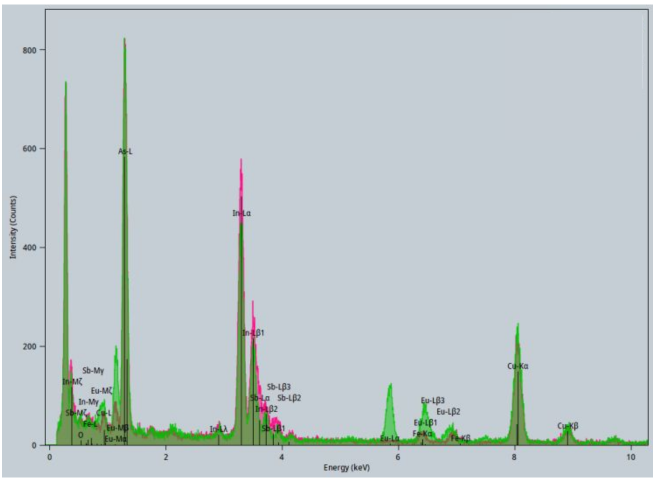

d

Area 1 - InAsSb

element	family	atomic fraction (%)
As	K	46.7 ± 3.0
In	L	49.0 ± 2.9
Sb	L	3.9 ± 0.5
Eu	L	0.3 ± 0.04

Area 2 - EuInAS

element	family	atomic fraction (%)
As	K	48.8 ± 2.7
In	L	39.6 ± 3.0
Sb	L	0 ± 0.04
Eu	L	11.7 ± 1.3

Figure SI(12): Elemental composition of a core/shell NWs with a shell of (EuIn)As grown on InAsSb. The data were obtained from a cross-sectional TEM sample viewed along the <111> growth direction. (a) HAADF image of the cross section, with the Eu rich shell concentrating to the left. (b) EDS elemental maps showing the distribution of In, Sb, Eu. Sb is present in the core, in the shell Eu replaces In. (c) Cumulated EDS spectra extracted from the two areas, Area 1 and Area 2, in (a). While the As signal remains constant, there is the opposite trend with respect to the In and Eu concentration, clearly observed in the intensity ratio between the In-L and the Eu-L lines. (d) Quantification of the spectra in (c). The Eu content in the shell is up to 12 at %, at the expense of the In content.

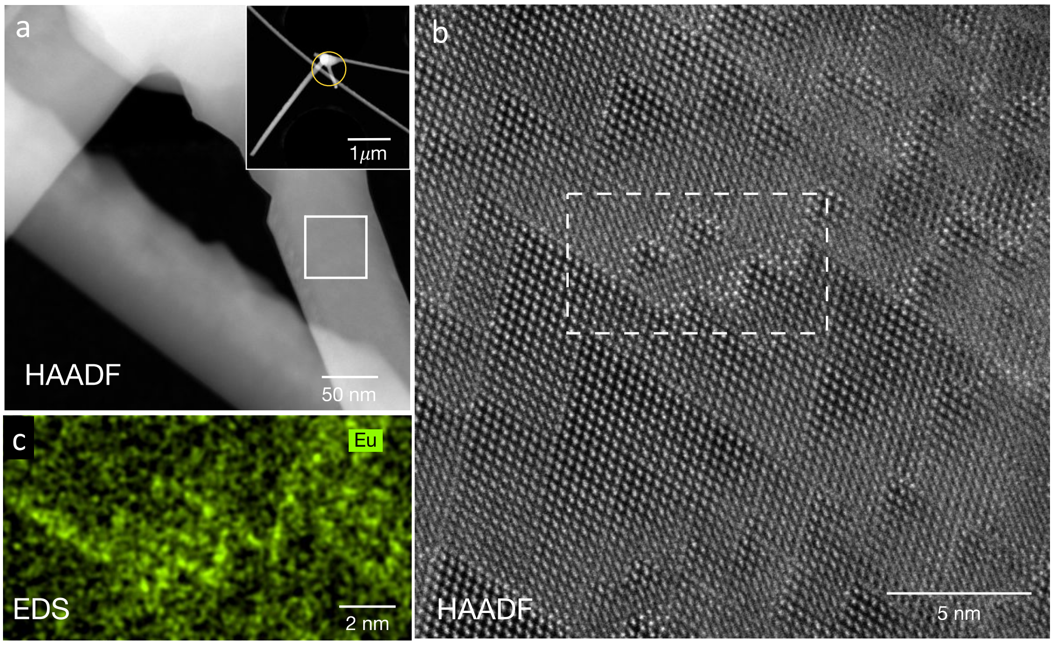

Figure SI(13): Nanodomain structure in stalactite ZB (EuIn)As NW grown from the intersection of two NWs grown on {111} plane. (a) HAADF images of the intersection of the NW from which <001> growth is observed. (b) Mosaic structure of (EuIn)As grown along the <001> direction of a stalactite NW. The image shows the region marked by a white triangle in (a) in atomic resolution. The typical triangular contrast of the grain boundary network is evident. (c) EDS elemental map of Eu, corresponding to the hatched rectangle in (b). The triangular domains boundaries are grain boundaries with Eu segregation.

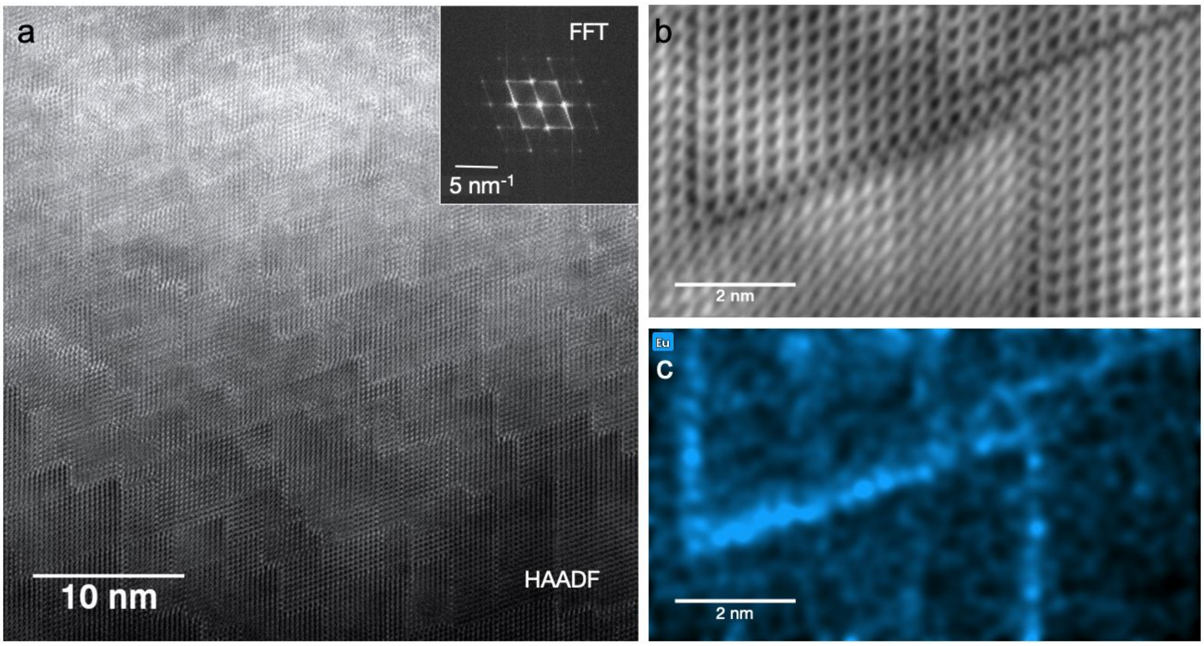

Figure SI(14): HAADF image taken from an (EuIn)As/InAs$_{1-x}$Sb$_x$ NW showing the typical mosaic structure and respective FFT (a) with a zoom in image (b) and related Eu mapping (c).

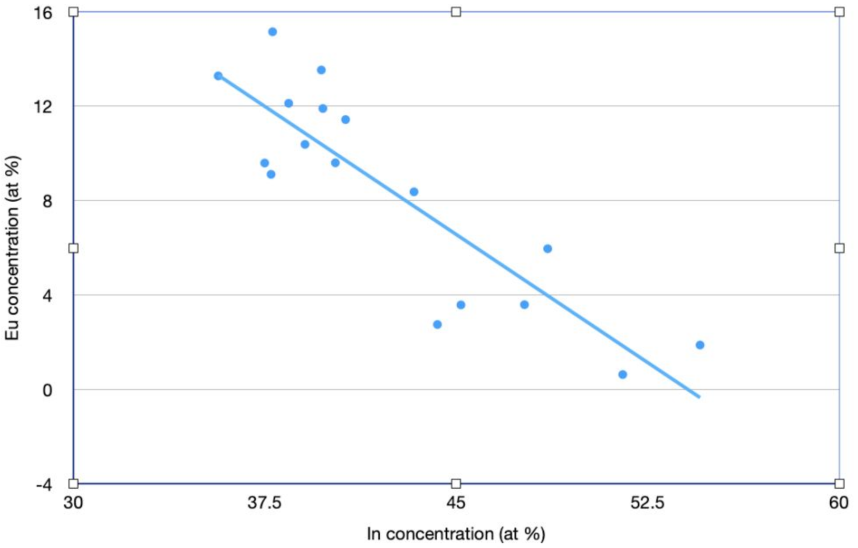

Figure SI(15): Indium substitution by europium. Scatter plot of atomic percentages of Eu versus In for various (EuIn)As NWs measured by STEM-EDS. The linear trend of the reduction of In content upon increase in Eu content is obvious. At the same time the As content stays nearly constant at 50 at %, so that In is essentially substituted by Eu.

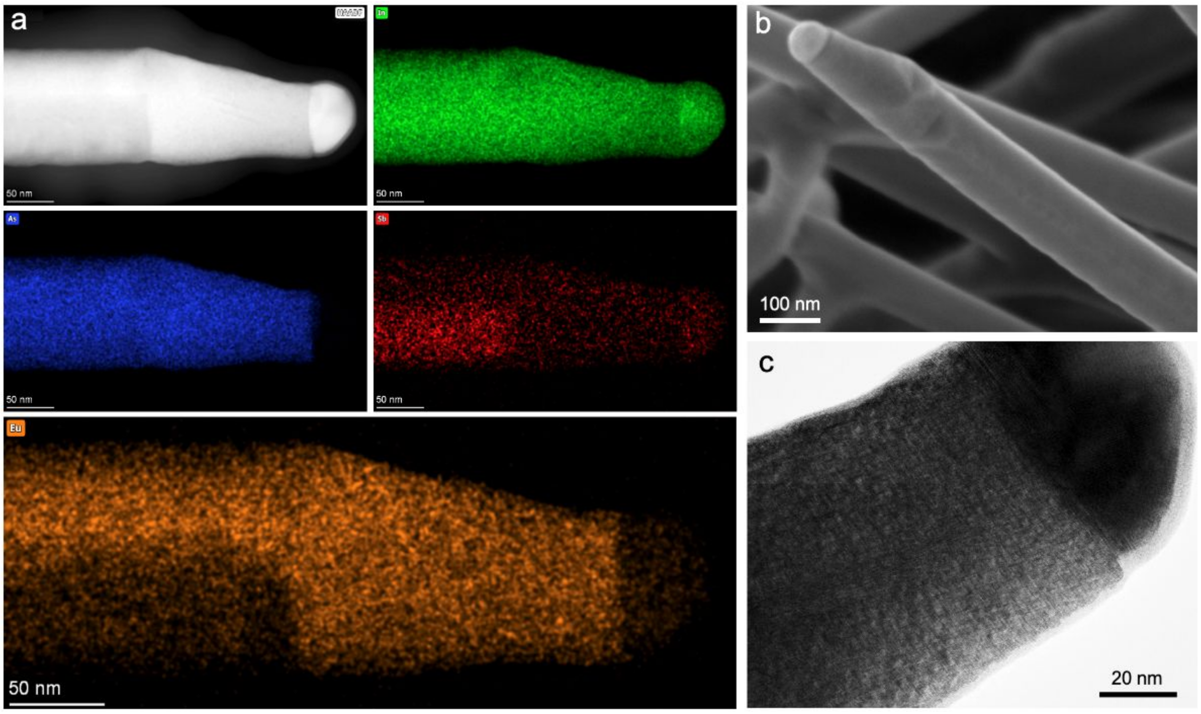

Figure SI(16): EDS data (a) of a sharpened tip (EuIn)As/InAs$_{1-x}$Sb$_x$ NW seen in the SEM image in (b) and HR-TEM image in (c). Eu composition of the tip is uniform at the tip, displaying the short axial growth which occurred during the growth of the (EuIn)As shell.

SQUID measurements

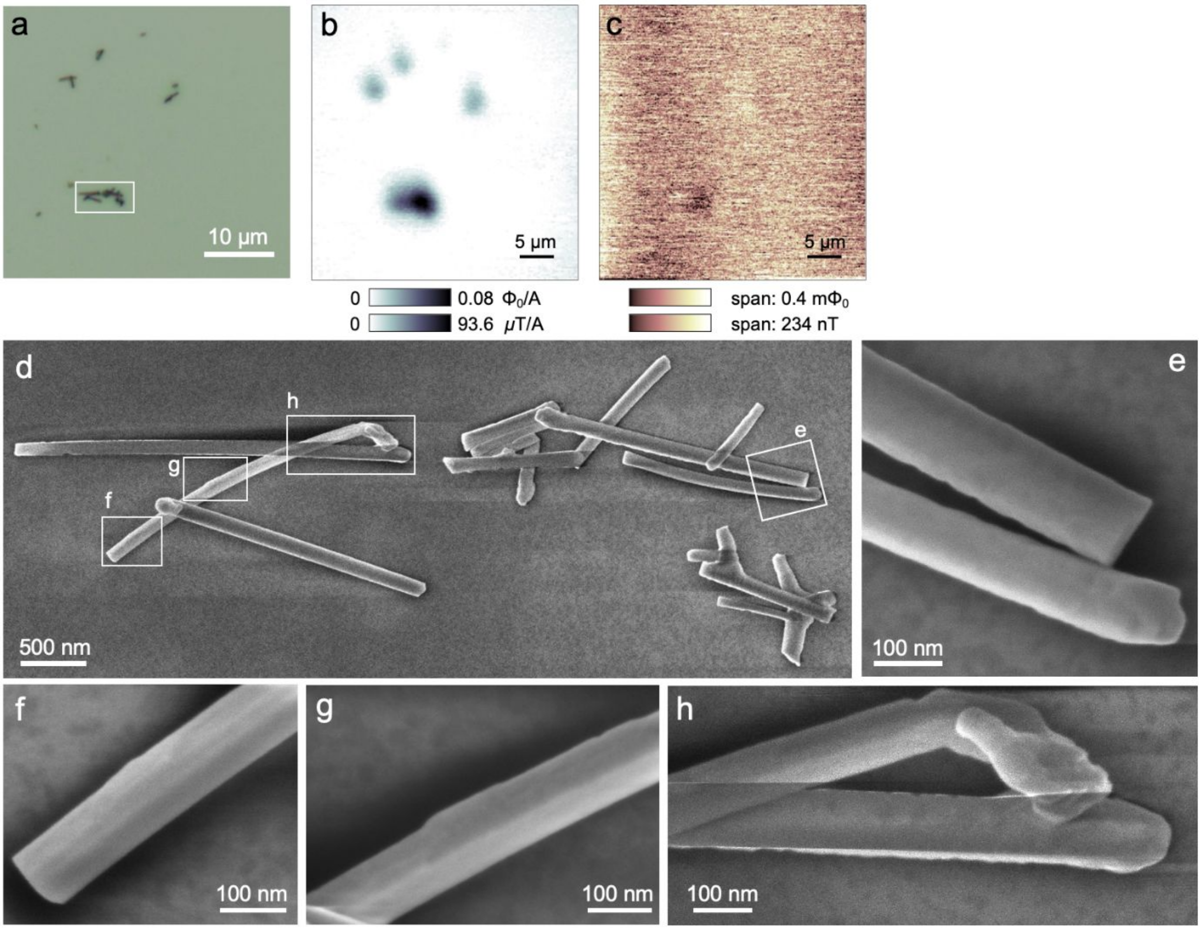

Figure SI(17): Scanning SQUID images of (EuIn)As/$InAs_{1-x}Sb_x$ NWs on a Si/SiO_2 substrate. Optical image (a), susceptibility map (b) and a map of the static magnetic landscape (c) of the NWs. (d-h) SEM images of NWs chosen from (a) and measured magnetically in (b-c)

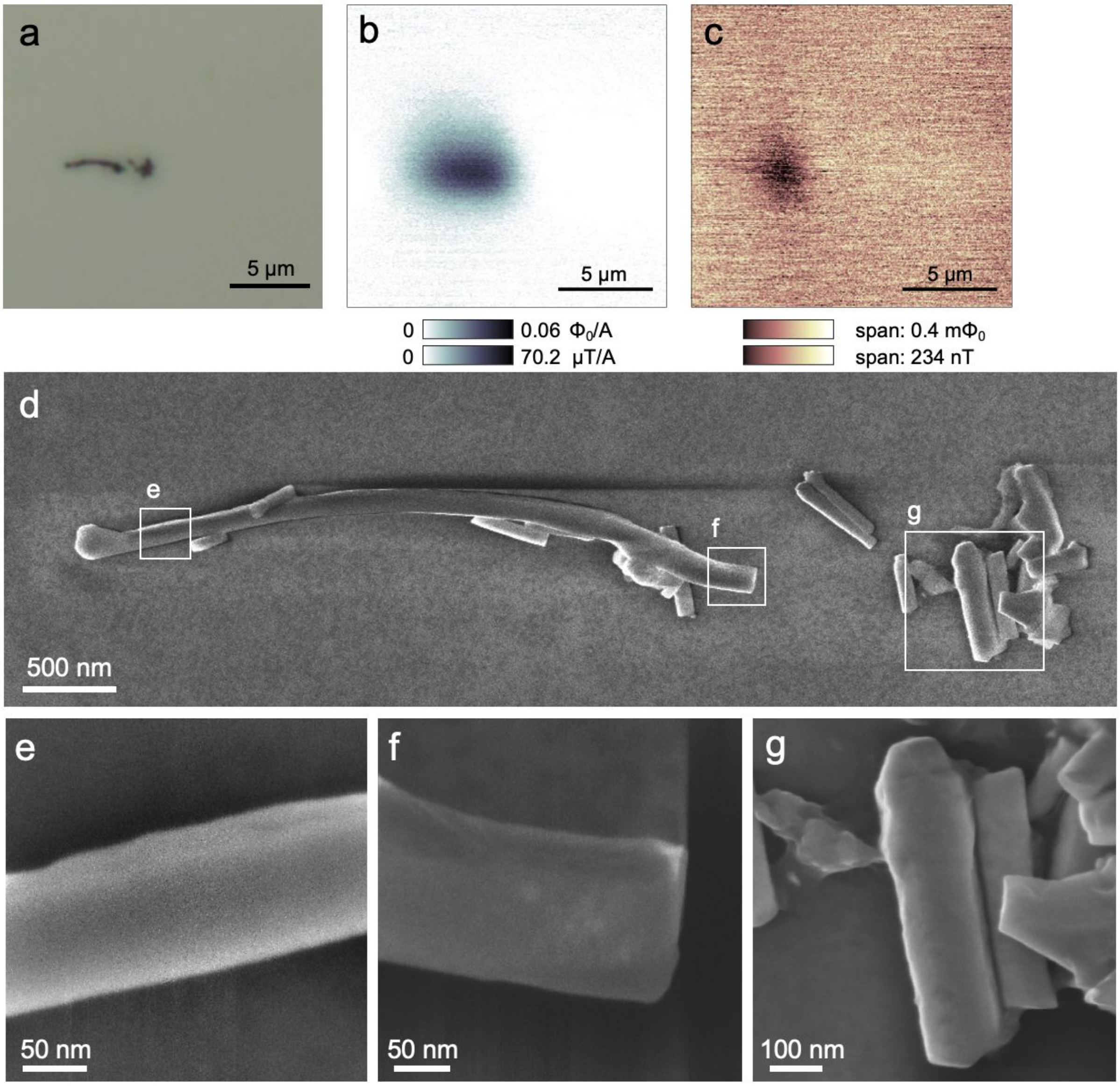

Figure SI(18): Scanning SQUID images of (EuIn)As/$InAs_{1-x}Sb_x$ NWs on a Si/SiO_2 substrate. Optical image (a). Susceptibility map (b) and a map of the static magnetic landscape (c) of the NWs. (d-h) SEM images of the NWs.

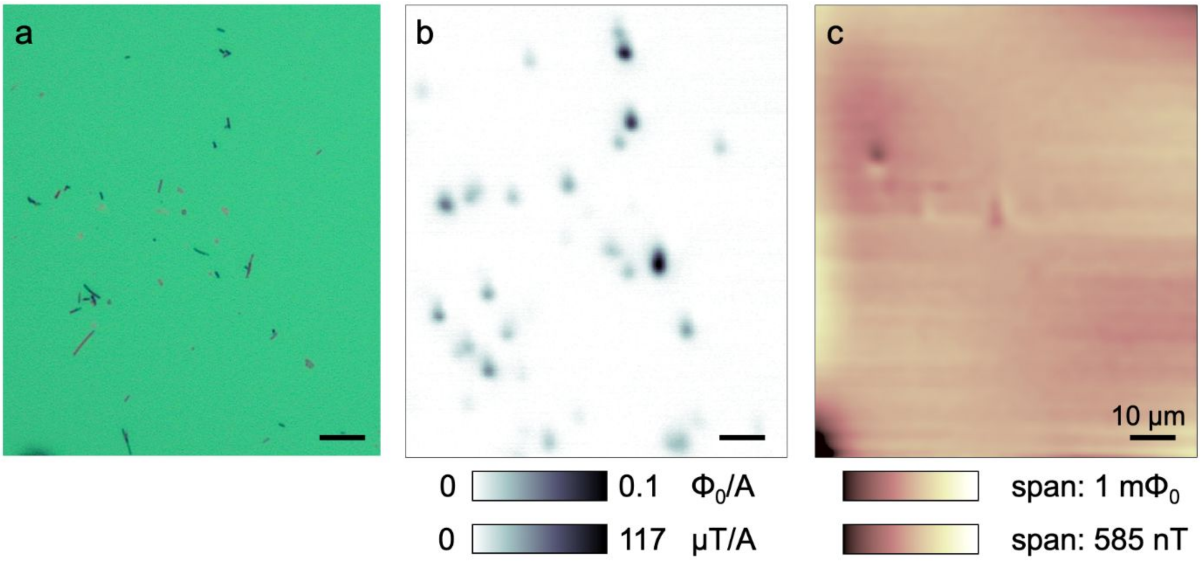

Figure SI(19): (a) An optical image showing large number of (EuIn)As/$InAs_{1-x}Sb_x$ NWs. (b) Susceptibility map, demonstrating detectible signal for most of the NWs, at various signal strengths. (c) A map of the static magnetic landscape.

Reference:

(1) Kang, J.-H.; Cohen, Y.; Ronen, Y.; Heiblum, M.; Buczko, R.; Kacman, P.; Popovitz-Biro, R.; Shtrikman, H. Crystal Structure and Transport in Merged InAs Nanowires MBE Grown on (001) InAs. *Nano Lett.* **2013**, *13* (11), 5190–5196. https://doi.org/10.1021/nl402571s.

(2) Kang, J.-H.; Galicka, M.; Kacman, P.; Shtrikman, H. Wurtzite/Zinc-Blende 'K'-Shape InAs Nanowires with Embedded Two-Dimensional Wurtzite Plates. *Nano Lett.* **2017**, *17* (1), 531–537. https://doi.org/10.1021/acs.nanolett.6b04598.

(3) Kang, J.-H.; Krizek, F.; Zaluska-Kotur, M.; Krogstrup, P.; Kacman, P.; Beidenkopf, H.; Shtrikman, H. Au-Assisted Substrate-Faceting for Inclined Nanowire Growth. *Nano Lett.* **2018**, *18* (7), 4115–4122. https://doi.org/10.1021/acs.nanolett.8b00853.

(4) Clarke, J.; I. Braginski, A. *The SQUID Handbook: Fundamentals and Technology of SQUIDs and SQUID Systems, I*; John Wiley & Sons, Ltd, 2004.

(5) Persky, E.; Sochnikov, I.; Kalisky, B. Studying Quantum Materials with Scanning SQUID Microscopy. *Annual Review of Condensed Matter Physics* **2022**, *13* (1), 385–405. https://doi.org/10.1146/annurev-conmatphys-031620-104226.

(6) Xu, T.; Dick, K. A.; Plissard, S.; Nguyen, T. H.; Makoudi, Y.; Berthe, M.; Nys, J.-P.; Wallart, X.; Grandidier, B.; Caroff, P. Faceting, Composition and Crystal Phase Evolution in III–V Antimonide Nanowire Heterostructures Revealed by Combining Microscopy Techniques. *Nanotechnology* **2012**, *23* (9), 095702. https://doi.org/10.1088/0957-4484/23/9/095702.

(7) Ercolani, D.; Gemmi, M.; Nasi, L.; Rossi, F.; Pea, M.; Li, A.; Salviati, G.; Beltram, F.; Sorba, L. Growth of InAs/InAsSb Heterostructured Nanowires. *Nanotechnology* **2012**, *23* (11), 115606. https://doi.org/10.1088/0957-4484/23/11/115606.

(8) Goforth, A. M.; Klavins, P.; Fettinger, J. C.; Kauzlarich, S. M. Magnetic Properties and Negative Colossal Magnetoresistance of the Rare Earth Zintl Phase EuIn2As2. *Inorg. Chem.* **2008**, *47* (23), 11048–11056. https://doi.org/10.1021/ic801290u.
